# Exploring the partitioning of hydrophobic organic compounds between water, suspended particulate matter and diverse fish species in a German river ecosystem

**DOI:** 10.1186/s12302-022-00644-w

**Published:** 2022-08-05

**Authors:** Theo Wernicke, Elisa Rojo-Nieto, Albrecht Paschke, Claudia Nogueira Tavares, Mario Brauns, Annika Jahnke

**Affiliations:** 1 Department of Ecological Chemistry, Helmholtz Centre for Environmental Research – UFZ, Permoserstr. 15, 04318 Leipzig, Germany; 2 Institute for Environmental Research, RWTH Aachen University, Worringerweg 1, 52074 Aachen, Germany; 3 Department of River Ecology, Helmholtz Centre for Environmental Research – UFZ, Brückstraße 3a, 39114 Magdeburg, Germany; 4 Department of Conservation Biology & Social-Ecological Systems, Helmholtz Centre for Environmental Research – UFZ, Permoserstr. 15, 04318 Leipzig, Germany

**Keywords:** Suspended particulate matter (SPM), Freshwater, Passive sampling devices (PSDs), Bioaccumulation, Hydrophobic organic compounds (HOCs), Fish, Proxy, Monitoring

## Abstract

**Background:**

Bioaccumulation of hydrophobic organic compounds (HOCs) along freshwater food chains is a major environmental concern as top predators in food webs are relevant for human consumption. To characterize and manage the associated risks, considerable numbers of organisms are sampled regularly for monitoring purposes. However, ethical and financial issues call for an alternative, more generic and more robust approach for assessing the internal exposure of fish that circumvents large variability in biota sampling due to interindividual differences. Passive sampling devices (PSDs) offer a fugacity-based approach for pollutant enrichment from different abiotic environmental compartments with a subsequent estimation of bioaccumulation in fish which we explored and compared to HOC concentrations in fish as determined using traditional approaches.

**Results:**

In this study, concentrations in silicone-based PSDs applied to the water phase and suspended particulate matter (SPM) of a river polluted with HOCs were used to estimate the concentration in model lipids at thermodynamic equilibrium with either environmental compartment. For comparison, muscle tissue of seven fish species (trophic level 1.8 to 2.8) was extracted using traditional exhaustive solvent extraction, and the lipid-normalized concentrations of HOCs were determined. The PSD-based data from SPM proved to be a more conservative estimator for HOCs accumulated in fish than those from water. Body length of the fish was found to be more suitable to describe increasing accumulation of HOCs than their trophic level as derived from stable isotope analysis and might offer a suitable alternative for future studies.

**Conclusions:**

By combining fugacity-based sampling in the abiotic environment, translation into corresponding concentrations in model lipids and body length as an indicator for increasing bioaccumulation in fish, we present a suggestion for a robust approach that may be a meaningful addition to conventional monitoring methods. This approach potentially increases the efficiency of existing monitoring programs without the need to regularly sacrifice vertebrate species.

**Graphical Abstract:**

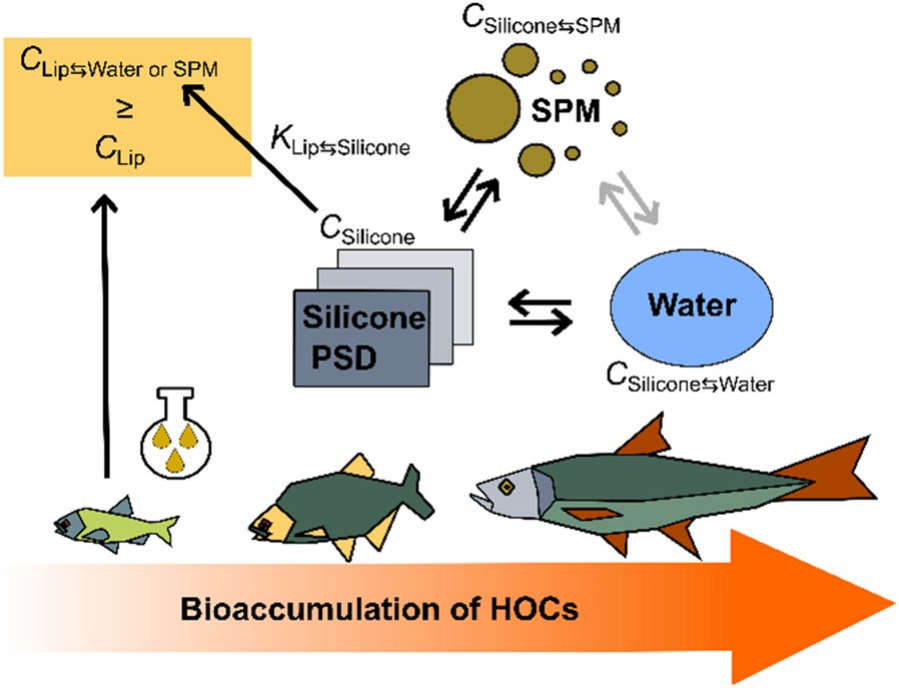

**Supplementary Information:**

The online version contains supplementary material available at 10.1186/s12302-022-00644-w.

## Introduction

Hydrophobic organic compounds (HOCs) tend to partition into nonpolar absorptive lipid-like materials. In aquatic environments, this tendency leads to the partitioning of HOCs into abiotic phases such as organic carbon (OC) in detritus, sediments and suspended particulate matter (SPM) and into biotic phases such as lipids of organisms. Driven by a gradient in fugacity, we often observe a passive net mass flux from the abiotic environment into the organism (bioconcentration), where HOCs accumulate mostly in lipid fractions of the tissue [1]. As organisms are organized in trophic networks, with a flux of energy from primary producers to primary consumers towards higher consumers, persistent HOCs may be biomagnified [2] with elevated fugacity in the higher trophic level (TL). Bioconcentration and biomagnification of a compound contribute to its bioaccumulation [3]. Whether primarily bioconcentration or biomagnification of HOCs is observed in an organism depends on the hydrophobicity of the compound and the TL of the organism, as well as its metabolic capacity. The accumulation of HOCs with a log *K*_OW_ < 5 in an organism is dominated by bioconcentration, whereas biomagnification via the digested food dominates the accumulation of more hydrophobic substances in organisms [2, 4, 5].

Increased, biomagnified concentrations of HOCs in an aquatic organism typically correlate positively with the TL of an organism [6], although trends are often not very clear due to high natural intraspecies and interspecies variability. Predators at high TL thus may show elevated biomagnification [6], which additionally increases with increasing hydrophobicity of the HOCs [7]. The ratio of stable nitrogen isotopes ^15^N to ^14^N in consumers’ tissue is traditionally used to characterize their TL in a food web, since ^15^N accumulates with increasing TL [8, 9].

In addition to their ecological role, adult predatory and omnivorous fish at the top of the food web in river ecosystems are also a relevant component of human diets [10, 11], underlining the need to understand the enrichment of HOCs in their tissues as pursued within comprehensive monitoring approaches. To assess the internal exposure of those fish, expressed as their lipid-normalized concentration *C*_Lip_, traditional monitoring programs have been conducted in many countries over the past decades [12, 13]. Substantial numbers of fish are regularly sacrificed to account for the large variability of physiological and chemical conditions in individual fish, differences between species as well as temporal and geographical trends. These regular monitoring programs are related to substantial cost and ethical issues.

In this context, an alternative, robust and cost-efficient monitoring approach free of ethical concerns is needed as a proxy to offer a less biased and more generic and robust estimation of the internal exposure of fish. Passive sampling devices (PSDs) applied in the different abiotic compartments surrounding the fish, such as sediment [14–16], SPM [17, 18], and water [19], have shown potential for this purpose, when the concentrations in the passive sampling polymer are translated into equilibrium partitioning concentrations in model lipids, *C *_Lip⇆Medium_. In general, PSDs can simulate the uptake of HOCs via bioconcentration into the lipid phase of low TL organisms [20]. To a certain extent, this simulation also appears valid in fish [21], where a positive log–log correlation was found between polymer mass-normalized concentrations of HOCs in PSDs and lipid-normalized concentrations in fish across different TLs.

PSDs have shown the potential to express the thermodynamic status of HOC partitioning between the environment and the lipid tissue of organisms [14, 15, 17, 19]. A suitable measure can be the *C*_Lip_/*C*_Lip⇆Medium_ ratio, also referred to as the partitioning status of lipids in organisms relative to their environment. The partitioning status proved to be a conservative estimator for the contamination of fish in similar previous work: Smedes et al. [19] showed that only *C*_Lip_ of fish of TL > 4 could be expected to reach or exceed *C*_Lip⇆Water_. In addition, *C*_Lip_ remained below *C*_Lip⇆Sediment_ for high trophic level predatory fish in a Swedish lake [14], as well as for non-piscivorous fish in the ecosystem of River Elbe [15]. Wernicke et al. [17] described PSDs equilibrated with SPM from diverse riverine systems in Germany as a mostly conservative proxy for non-piscivorous fish but found that fish of high age could exceed the partitioning status of 1 by up to a factor of 1.5 for very bioaccumulative compounds and very old individuals.

In this study, we investigated *C*_Lip_ of model HOCs such as polychlorinated biphenyls (PCBs) and organochlorine pesticides (OCPs) in a single species (European chub, *Squalius cephalus*) of diverse age sampled in 2017 from a lowland river formerly subjected to industrial effluents. Furthermore, we compared these results to diverse fish species from different ecological niches sampled in 2020 from the same location. We then used silicone-coated glass jars for *ex-situ* equilibrium passive sampling of SPM to determine *C*_Lip⇆SPM_, as well as silicone sheet PSDs for the *in-situ* kinetic sampling of water in the river (extrapolated to equilibrium using PRCs) to determine *C*_Lip⇆Water_ and thus assess the thermodynamic status between water and SPM. Finally, we compared *C*_Lip⇆SPM_ and *C*_Lip⇆Water_ of HOCs to *C*_Lip_ measured in the investigated fish sampled in 2020 to evaluate the performance of the PSD-based proxies as robust, low-cost monitoring approaches free of ethical concerns for future applications.

## Materials and methods

### Sampling area

Sampling was performed in the River Mulde near Dessau (Germany), 10–24 km upstream of its mouth to the River Elbe (51.731550, 12.295652, see map in Additional file 1: Figure S1), including the nature conservation area “Unteres Muldetal”. Fishing and angling are only allowed in a small section of the lower reach of the River Mulde, located 10 km downstream of our sampling site. Our sampling site and the surrounding floodplain belong to the biosphere reserve “Mittlere Elbe”, and we can thus exclude a potential bias induced by fish stocking. Due to legacy contamination from a former chemical production site near Bitterfeld, approx. 20 km upstream of the sampling area, the ecosystem of River Mulde is particularly exposed to legacy contamination by hexachlorocyclohexane (HCH) isomers, dichlorodiphenyltrichloroethane (p,p′-DDT) and its metabolites and chlorinated benzenes [22, 23], such as hexachlorobenzene (HCB) [22–24].

### Chemicals and analysis

Analytical standards used for data quantification consisted of 8 polychlorinated biphenyls (PCBs), 14 polycyclic aromatic hydrocarbons (PAHs), HCB, the alpha, beta, gamma and delta isomers of HCH, p,p′-DDT and its metabolites dichlorodiphenyldichloroethane (p,p′-DDD) and dichlorodiphenyldichloroethylene (p,p′-DDE). Stable isotope-labeled internal standards (IS) and performance reference compounds (PRCs) used in water PSDs are listed, among other details, in Additional file 1: Sect. 2 and Table S1.

Chemical analysis was performed by gas chromatographic separation and detection using tandem mass spectrometry, using a 7890A GC System coupled to a 7000 GC/MS TripleQuad (Agilent Technologies, USA). Quantification was based on matrix-method-matched calibrations. See more details in Additional file 1: Sect. 3 and Table S1.

### PSDs as a proxy for biota

Using sheets or coatings of a suitable reference polymer, such as silicone, PSDs can be applied as a proxy to estimate the fugacity gradients of HOCs in the abiotic environment [25, 26]. For sampling in equilibrium mode (sediment, SPM), the HOCs in the PSDs equilibrated with the environmental compartment of interest are then extracted and quantified, yielding the polymer mass-normalized equilibrium concentration *C*_Polymer_. For kinetic sampling (water), these *C*_Polymer_ usually need to be extrapolated to equilibrium as derived from the dissipation of PRCs during deployment (see Sect. 2.7.1) [27] unless reliable sampling rates are available. At thermodynamic equilibrium, the fugacity of a compound in a PSD equals the fugacity in the sampled medium. *C*_Polymer_ then can be translated into a model lipid reference phase allowing comparisons with *C*_Lip_ in biota. For all cases, *C*_Polymer_ is then multiplied with compound-specific lipid/polymer partition coefficients *K*_Lip/Polymer_ to derive concentrations in model lipids at thermodynamic equilibrium by the medium (water, SPM or sediment) *C*_Lip⇆Medium_. This generic measure *C*_Lip⇌Medium_ then corresponds to the concentration in model lipids at equilibrium with either water *C*_Lip⇆Water_, SPM *C*_Lip⇆SPM_ or sediment *C*_Lip⇆Sediment_. *C*_Lip⇆Water_, *C*_Lip⇆SPM_ and *C*_Lip⇆Sediment_ can finally be compared to actual *C*_Lip_ of HOCs in fish from the same environment and sampling time frame as determined by traditional exhaustive solvent extraction followed by normalization to the lipid fraction, to evaluate the performance of the proxies.

### *In-situ* passive sampling in surface water

Passive sampling in the aqueous phase followed the guideline provided by Smedes and Booji [28]. As PSD material, we used the silicone SSP-M823 (Dow Corning, USA) of 350 µm thickness. The sheets were cut into pieces of 50 × 100 mm corresponding to 1.9 ± 0.4 g and cleaned by Soxhlet extraction with ethylacetate for ~80 h, soaked in 500 mL of methanol in an Erlenmeyer flask to remove the ethylacetate and transferred to a fresh 500 mL aliquot of methanol in a 2 L amber glass jar with an aluminum-lined screw cap for storage until further processing. As described in Smedes and Booji [28], 25 ng of six selected PRCs, that covered the range of physicochemical properties of the analytes of interest, were spiked to each PSD replicate (one replicate consisting of four silicone sheets). The depletion of PRCs from deployed PSD replicates when exposed to the sampled water body was used to characterize the thermodynamic equilibration status of each PSD replicate towards the water phase. For the analytical quantification of the field samples, ten replicates were used to create a method-matched calibration. Three replicates were used as laboratory controls, and another three replicates as field controls.

For field sampling, four sets of sheets (i.e., 12 individual sheets) were deployed for 28 days (August 10, 2020 to September 07, 2020) using a continuous flow-through stainless steel box (30 cm × 40 cm × 60 cm; water-volume 72 L) covered with a lid to prevent contamination from dust and aerosols. The box had an in- and outlet of 2 cm diameter. It was placed in a monitoring station in Dessau operated by the Saxony-Anhalt Federal State authority for flood protection and water management (Landesbetrieb für Hochwasserschutz und Wasserwirtschaft, LHW) to prevent vandalism. Fresh, unfiltered river water was constantly pumped through the box with a flow of ~ 300 L/h, ensuring that the water temperature corresponded to the temperature in the river. After the 28-day deployment, the silicone sheets were gently rubbed with bare hands in the river water to remove any biofilm from their surfaces. The samples (three sheets each pooled together) were then dried with lint-free paper tissues, wrapped into hexane-rinsed, dried aluminum foil and placed sample-specifically in ziplock bags for immediate storage at − 20 °C. During the time of handling, including the duration of the installation, cleaning and collection of the samplers, all field controls were exposed to the surrounding air at the deployment site and transported and stored in the same way as the samplers. While the actual samples were deployed in the field, field controls were stored at − 20 °C in the laboratory freezer.

In the laboratory, the deployed samplers, field controls and lab controls were dried with lint-free tissues. All sheets were weighed before extraction. Every sample consisting of three individual sheets was placed in a 120 mL glass jar with 30 mL of ethylacetate (ca. 1 mL per 0.1 g silicone), spiked with 20 µL of a 1000 pg/µL IS solution and rolled for 2 h on a roller mixer according to pre-established protocols. A piece of hexane-rinsed, dried aluminum foil was placed inside the lid of each jar to prevent contact of the ethylacetate with the lining. The extraction process was repeated once without the addition of new IS. The combined extract was reduced under a nitrogen stream, transferred to 1.5 mL acetonitrile and cleaned over 3 mL Captiva EMR-Lipid cartridges (Agilent Technologies, USA) [29]. The extracts were then blown down to ~5 µL and dissolved in 100 µL ethylacetate for chemical analysis.

A method-matched calibration was prepared in the same way as the samples were processed, but spiked with the IS at the same level and an analytical standard mix as well as the PRC mix at diverse appropriate levels to yield the following concentrations in the final 100 µL ethylacetate extracts: 200 pg/µL for the IS and 0.1, 0.5, 1, 10, 20, 50, 100, 200, 500, and 1000 pg/µL for the native compounds and PRCs.

### *Ex-situ* passive sampling from suspended particulate matter

Freeze-dried SPM samples collected with a sedimentation trap [30] and pooled over single months (April, May, June, July and August 2020) were provided by the LHW, including their measured TOC contents (see Additional file 2: Table S8). Passive sampling from SPM was conducted separately for each sampled month, and monthly data were used for further analysis. Raw river water for the sedimentation trap was pumped continuously from a water depth of ~ 50 cm. Due to the dynamic character of the River Mulde, we assumed mixed conditions and hence an equal distribution of SPM across the water column.

Passive sampling from SPM was done in silicone-coated glass jars [25] and followed the protocol described for sediment [25, 31, 32] in a modified approach [17]. Briefly, 10 g of freeze-dried SPM and 40 mL of bi-distilled water were rolled at room temperature in silicone-coated jars (silicone used: Dowsil DC 1–2577, Dow Chemical Company, USA) with coating thicknesses (average silicone mass) of 1 (7.7 mg), 2 (16.4 mg), 4 (24.7 mg), 8 (62.1 mg) and 16 µm (100.7 mg) for 3 weeks. After removing the sample, rinsing with bi-distilled water and thorough surface wiping using lint-free tissues, the chemicals in the silicone coating were extracted twice with ethyl acetate, with IS spiked to the first aliquot. Depleting the SPM sample by the silicone coating by more than 5% of the total chemical content had to be avoided [33]. Depending on the OC mass in the SPM samples and compound-specific properties, certain coating thicknesses were thus excluded from further data analysis (see Additional file 1: Sect. 6 for details).

### Field sampling of fish and exhaustive extraction of their muscle tissues

Our field sampling of fish had an opportunistic character, where we focused only on fish of considerable size. We assumed that all fish are exposed to sources of HOCs from water or SPM in a similar way, except for benthic feeders that might have a higher exposure to HOCs from deposited SPM. Fish of different species were sampled using a combination of electro-fishing and gillnets on September 7–10, 2020, as part of a larger project addressing the effects of river restoration on ecological parameters [34]. Ethical approval for our studies had been granted by the local environmental agency. Individuals were taxonomically identified, and individual body length and body weight were measured. Individuals of >20 cm in body length, suitable for the dissection of the muscle tissue, were chosen for further processing in the field.

Individuals were taxonomically identified, euthanized with a blow to the head, weighed and measured in length (tip of the head to tip of the caudal fin), and the dorsal muscle tissue was dissected. The tissue sample was then wrapped in aluminum foil and put on ice for at max. 6 h before freezing at −20 °C. Cutting surfaces and tools were cleaned with ethanol between samples to avoid cross-contamination. Species sampled were: Eel (*Anguilla anguilla*) (*n* = 2), Ide (*Leuciscus idus*) (*n* = 4), European perch (*Perca fluviatilis*) (*n* = 3), European chub (*n* = 5), Pike (*Esox Lucius*) (*n* = 4), Asp (*Leuciscus aspius*) (*n* = 1) and catfish (*Silurus glanis*) (*n* = 2). The body length and weight of all sampled fish can be found in Table 1 and Additional file 2: Table S8.

**Table 1:** Characteristic data of chub samples from 2017 and diverse species sampled in 2020, regarding the number of sampled individuals, total body length, total weight, the lipid content of the extracted muscle tissue and trophic level

Species	Number of individuals	Total body length mean ± sd (cm)	Total body length range (cm)	Total weight mean ± sd (g)	Lipid content mean ± sd (%)	Trophic level mean ± sd
Chub (2017)	20	23.8 ± 13.4	11.0–53.0	403 ± 673	1.5 ± 0.8	2.5 ± 0.2
Chub (2020)	5	52.1 ± 3.7	46.6–56.5	1250 ± 360	2.0 ± 0.04	2.4 ± 0.4
Ide	4	49.7 ± 1.5	48.0–52.0	2060 ± 174	3.6 ± 0.9	2.3 ± 0.14
Perch	3	21.8 ± 1.2	20.5–23.0	142 ± 22.8	0.8 ± 0.01	2.6 ± 0.03
Eel	2	56.0 ± 4	52.0–60.0	305 ± 45.0	7.7 ± 2.8	2.3 ± 0.02
Pike	4	31.4 ± 1.2	30.0–33.0	121 ± 23.5	0.7 ± 0.07	2.7 ± 0.07
Asp	1	61		1580	0.2	2.6
Catfish	2	60.6 ± 20.2	51.0–103	2210 ± 2760	0.6 ± 0.02	2.5 ± 0.2

Biomagnification, as derived from measured HOC concentrations, may not only vary with the species and its TL, but also with age as approximated by individual body length, nutritional status, or general condition. We thus analyzed if the intraspecies HOC concentrations varied with body length and TL using archived samples of a single species: European chub. These samples (*n* = 20) were collected in 2017 in the context of the “Wilde Mulde” project from the same location in an identical way and were processed as described for the other samples collected in 2020.

In the laboratory, muscle tissue samples were thawed and homogenized using a laboratory-grade blender (1.2 L 8011EG, Waring, USA). Subsamples of 450 to 926 mg of the homogenized tissues were extracted using the “modified II” method (Jensen et al. [35] as described in detail by Wernicke et al. [17]). Briefly, tissue samples were extracted in three steps with mixtures of 2-propanol, diethyl ether and *n*-hexane in different compositions. The extracted lipid mass was noted. The extract was prepared for chemical analysis in 100 µL of ethylacetate.

### Quality assurance and quality control

Quantification of all analyzed chemicals was conducted using a method-matrix-matched calibration, following the identical extraction and clean-up procedures as for the samples. Preparation controls of passive water samplers (*n* = 3), laboratory control blanks (*n* = 3) of the extraction of fish muscle and laboratory control blanks (*n* = 4) of passive sampling of SPM formed the basis for the calculation of the respective method detection/quantification limits (MDL/MQL, see Additional file 1: Sect. 4). Only data points with concentrations above the MQL were used for further data analysis, interpretation and presentation. For passive samplers in water, compounds that did not exceed ten times the average *C*_Polymer_ found in the field control silicone sheets were excluded from further analysis [28].

### Calculations and statistical processing

#### Passive samplers in surface water

The aim of the passive sampling in the river water was to determine the HOC concentrations in PSDs following their exposure to the river water, to extrapolate to equilibrium partitioning concentrations based on the dissipation of multiple PRCs and the subsequent translation into equilibrium partitioning concentrations in model lipids at equilibrium with the water for the analyzed HOCs. This equilibration is challenging to achieve for HOCs in the water phase, if not impossible, in a reasonable time frame for very hydrophobic compounds (log *K*_OW_ >6) [26]. Therefore, the degree of equilibrium (DEQ) that the silicone sheets had reached with the water was characterized using the depletion of the spiked PRCs, covering a range of hydrophobicity. The DEQ for each target substance can be determined using these data with their assigned PRC. Calculations followed the guideline by Smedes and Booji [28]: The retained PRC fraction *f* was calculated for each PRC by the ratio of the amount left in the field sampler *N*_t_ upon retrieval from the field and the measured original amount as derived from the laboratory controls (average) *N*_0_ (Eq. 1):

1 $$\text{f } \, =\frac{{N}_{t}}{{N}_{0}}$$

*f* can also be expressed as follows (Eq. 2):

2 $$\text{f } \, ={ e}^{- \frac{B\times t}{{K}_{pw}\times {M}^{0.47}\times m}}$$

where *B* is a constant depending on the hydrodynamic condition around the deployed sampler, *t* is the deployment duration, *K*_pw_ is the compound-specific polymer/water partition coefficient for the sampler, *M* is the molar mass and *m* is the mass of the sampler.

B can be obtained by fitting Eq. (2) with an unweighted non-linear least-squares function for *f* against *K*_pw_M^0.47^ for each sampler replicate using the statistical programming environment R [36]. The sampling rate *R*_s_ for each compound can then be calculated as follows (Eq. 3):

3 $${R}_{s} =\frac{B}{{M}^{0.47}}$$

The DEQ was calculated according to Vrana et al. [26] (Eq. 4):

4 $${\text{DEQ}} = \left(\text{1}-{ e}^{- \frac{{R}_{s}\times t}{ {K}_{pw}\times m}}\right)$$

To cover the error propagation from estimation uncertainties (standard error) of the non-linear models for *B* and *K*_pw_ (taken from Smedes [37]), a first-order Taylor series error propagation method was conducted to estimate the uncertainties for DEQ [38]. We then derived the concentrations in silicone at equilibrium with the water phase *C*_Polymer⇆Water_ from the measured *C*_Sil_ (Eq. 5):

5 $${C}_{\mathrm{Polymer}\leftrightarrows \mathrm{Water}} =\frac{{C}_{\mathrm{Polymer}}}{\mathrm{DEQ}}$$

#### Partitioning status of fish

To characterize fugacity gradients between environmental compartments, we use the partitioning of HOCs into model lipids as a reference phase for water and SPM. Passive sampler-derived equilibrium partitioning concentrations in the silicone at equilibrium with water *C*_Sil⇆Water_ or SPM *C*_Sil⇆SPM_ can be converted into equilibrium partitioning concentrations in model lipids at equilibrium with the respective environmental compartment *C*_Lip⇆SPM_ or *C*_Lip⇆Water_. To obtain those data points, we multiplied *C*_Sil⇆Water_ with lipid/silicone partition coefficients for SSP (*K*_Lip/SSP_) [39] (Eq. 6) or *C*_Sil⇆SPM_ with the corresponding partition coefficients for DC1-2577 (*K*_Lip/DC_) (taken from the literature [40] or calculated, see Text Additional file 1: Sect. 6 for details) (Eq. 7):

6 $${C}_{\mathrm{Lip}\leftrightarrows \mathrm{Water}} ={C}_{\mathrm{Sil}\leftrightarrows \mathrm{Water}}\times {K}_{\mathrm{Lip}/\mathrm{SSP}}$$

7 $${C}_{\mathrm{Lip}\leftrightarrows \mathrm{SPM}} ={C}_{\mathrm{Sil}\leftrightarrows \mathrm{SPM}}\times {K}_{\mathrm{Lip}/\mathrm{DC}}$$

The measured lipid-normalized concentration from the exhaustive extraction of fish *C*_Lip_ and *C*_Lip⇆Water_ or *C*_Lip⇆SPM_ were then used to calculate the partitioning status of fish relative to either water or SPM (Eq. 8):

8 $$\text{Partitioning status }=\frac{{C}_{\mathrm{Lip}}}{{C}_{\mathrm{Lip}\leftrightarrows \mathrm{Water}}{\; {\text{or}} \; C}_{\mathrm{Lip}\leftrightarrows \mathrm{SPM}}}$$

PAHs were excluded from partitioning status calculations in the main manuscript due to extensive metabolism by fish that renders the assessment of thermodynamic characteristics for those compounds too complex for the scope of this study [41]. For details on the quantified PAHs, see Additional file 1: Sect. 9 and Figure S9.

#### Stable isotope analysis and trophic level

For details on stable isotope analysis, see Additional file 1: Sect. S7. The TLs of fish can be calculated (Eq. 9) by relating their δ^15^N values to a trophic baseline derived from stable isotope values of macroinvertebrate primary consumers (i.e., Chironomidae, *Corbicula fluminea*, *Dreissena polymorpha*, *Heptagenia* sp., Tipuliidae):

9 $$\text{TL} =\left(\frac{{\delta }^{15}{\text{N}}_{\text{Fish}}-{\delta }^{15}{\text{N}}_{\text{Primary Consumer}}}{{\Delta }^{15}\text{N}}\right)+2$$

assuming a trophic discrimination factor (Δ^15^N) of 3.4‰ [42], with *δ*^15^N_Fish_ being the stable nitrogen isotope ratio measured in the fish samples [43].

To identify different isotope sources which define the TL of fish, Post [43] suggested calculating weighted TLs based on a pelagic and a benthic food source. For this study, this calculation was done (Eq. 10) by

10 $${\text{TL}}= \left(\frac{{\delta }^{15}{N}_{\text{Fish}}-{(\delta }^{15}{N}_{\text{Benthic Primary Consumer}}* \alpha +{\delta }^{15}{N}_{\text{Pelagic Primary Consumer}}*(1-\alpha ))}{{\Delta }^{15}N}\right)+2$$

where *δ*^15^N_Benthic Primary Consumer_ = 15.37 per mil is the mean *δ*^15^N of Chironomidae and *Heptagenia flava*, δ^15^N_Pelagic Primary Consumer_ = 14.91 per mil is the mean δ^15^N of *Corbicula fluminea,* and *Dreissena polymorpha* (Additional file 1: Tables S4 and S5, Brauns et al., unpublished data) and α is the proportion of nitrogen derived from the primary consumer base of the food web. It was calculated (Eq. 11) as

11 $$\alpha = \frac{{\delta }^{13}{C}_{\text{Fish}}-{\delta }^{13}{C}_{\text{Pelagic Primary Consumer}}}{{\delta }^{13}{C}_{\text{Benthic Primary Consumer}}-{\delta }^{13}{C}_{\text{Pelagic Primary Consumer}}}$$

## Results and discussion

### Passive sampling in surface water

**Fig. 1:**
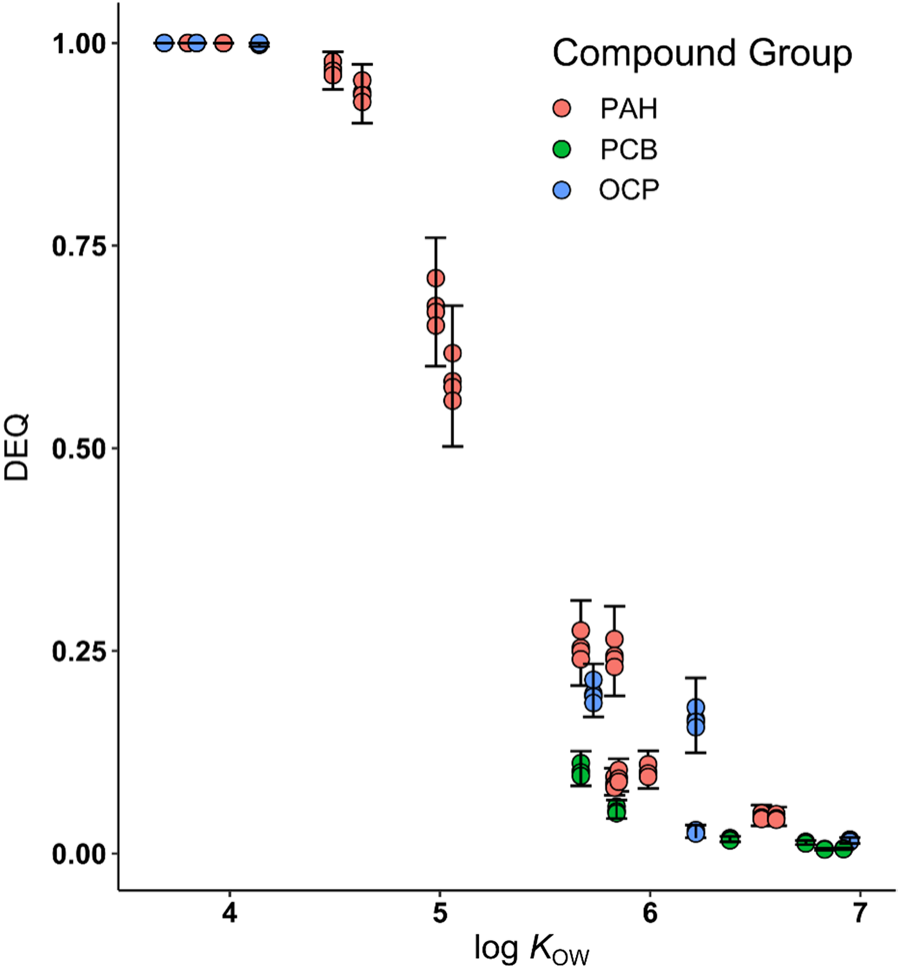
Degree of equilibrium (DEQ) against the log *K*_OW_ of the analyzed HOCs grouped into PAHs, PCBs and OCPs for silicone sheets used as water passive samplers. Error bars encompass the accumulated standard error of the four PSD replicates for each compound

The mean sampling rate *R*_s_ calculated using Eq. (3) ranged from 2.9 L/day for DDE to 4.9 L/day for Acenaphthylene. The mean DEQ calculated according to Eq. (4) ranged from 0.005 for PCB 138 (i.e., < 1% of equilibrium reached) to 1.0 (i.e., 100% equilibration) for beta HCH, gamma HCH, delta HCH, Acenaphthene, Acenaphthylene and Fluorene (Fig. 1), see Additional file 2: Table S8 for details. The maximum log *K*_OW_ for compounds reaching 95% of equilibrium was 4.6 (Anthracene). Vrana et al. [26] reported that 95% of equilibrium was reached for compounds up to a log *K*_OW_ of 5.5 (SSP silicone sheet of 500 µm thickness, deployed at a fish pond discharge, mean water temperature: 21.9 °C, this study: 24.4 °C) within 134 days, which is considerably longer than the 28 days deployment time in our study. The DEQ for the analyzed individual compounds according to their compound classes in this study is shown in Fig. 1. Very low DEQ values for HOCs of a log *K*_OW_ >5.5 (DEQ mean values ranged from 0.25 for Chrysene to 0.005 for PCB 138) potentially imply larger uncertainties for estimating *C*_Lip⇆Water_ for those compounds due to the extrapolation from a low DEQ to 100% equilibrium.

The lowest *C*_Lip⇆Water_ (mean ± standard deviation) was estimated for PCB 28 at 6.1 ± 0.2 pg/mg and the highest for DDT at 2530 ± 930 pg/mg. See also Additional file 1: Table S6.

### Passive sampling from suspended particulate matter

For the passive sampling from SPM, the range of mean *C*_Lip⇆SPM_ quantified out of the coated jars with up to five coating thicknesses (depletion avoidance criteria were applied, and some outliers were removed as described in detail in Additional file 2: Table S9) encompassed PCB 28 at 175 ± 61 pg/mg to DDD at 23,900 ± 9030 pg/mg, see Additional file 2: Table S8 for details.

### Concentrations in fish muscle tissue

We focus in the following on five model HOCs (beta HCH, HCB, PCB 118, PCB 153 and DDE), which represent a wide range of hydrophobicity (log *K*_OW_ = 3.84 for beta HCH to log *K*_OW_ = 6.95 for DDE) and could be quantified in the majority of samples (data regarding other compounds can be found in Additional file 2: Table S8). Therefore, they offer a suitable basis for the comparison of different sample types.

*C*_Lip_ for the chub samples collected in 2017, where quantifiable, ranged for beta HCH from 11.3 pg/mg to 180 pg/mg, for HCB from 16.9 pg/mg to 178 pg/mg, for PCB 118 from 16.0 pg/mg to 99.1 pg/mg, for PCB 153 from 1.85 pg/mg to 430 pg/mg and for DDE from 106 pg/mg to 2270 pg/mg. *C*_Lip_ (mean ± standard deviation) for the 2020 samples for beta HCH ranged from 34.0 pg/mg in catfish to 226 ± 20.1 pg/mg in eel, for HCB from 43.1 ± 4 pg/mg in perch to 236 ± 4.1 pg/mg in eel, for PCB 118 from 21.6 ± 13.2 pg/mg in chub to 156 pg/mg in asp, for PCB 153 from 11.9 ± 9.5 pg/mg in perch to 1400 pg/mg in asp and for DDE from 38.5 pg/mg in perch to 2970 pg/mg in asp. *C*_Lip_ data are displayed in Additional file 1: Figure S8 plotted against the TL measured in European chub caught in 2017 (A) and diverse fish species collected in 2020 (B).

### Trophic relationships

The TL of individual chub from 2017 ranged from 1.94 to 3.06. The TL across all fish species sampled in 2020 ranged from a minimum of 1.78 for chub to a maximum of 2.81 for pike (see also Table 1). Since primary consumers are associated with TL = 2, the TL range for the fish from 2020 appears unrealistically low. A reason for this observation might be high variability in the *δ*^15^N values of invertebrates, serving as a reference for TL = 2 in our data set, possibly due to variable nitrogen sources from agriculture run-off. Log-linear models were created for each sampling year, comparing *C*_Lip_ of each HOC against individual TL. For both sampling groups, beta HCH and HCB showed no or only a weak relationship with TL, which could be expected, since those substances with relatively low hydrophobicities (log *K*_OW_ = 3.84 and 5.73) are known not to biomagnify in piscivorous fish (HCH) [44] or show some variation in different ecosystems (HCB) [45]. For chub from 2017, the models indicate a positive relationship of log *C*_Lip_ with increasing TL for DDE (*p* value = 0.044) and PCB 153 (*p* value = 0.032) (Additional file 1: Figure S8A), the most hydrophobic model chemicals (log *K*_OW_ = 6.95 and 6.92) discussed here. Similar relationships were also observed by Smedes et al. [19].

For the fish samples from 2020, the linear models indicated a negative relationship between log *C*_Lip_ and TL even for those very hydrophobic compounds, PCB 153 (*p* value = 0.139) and DDE (*p* value = 0.117) (Additional file 1: Figure S8B). These two findings for chub sampled in 2017 and diverse fish species collected in 2020 are in contrast with each other.

Walters et al. [45] suggested PCB 153 as a benchmark chemical for bioaccumulation. They found that the trophic magnification factor (TMF, see also Borgå et al. [6] for calculations) of PCB 153 had a range of 1.5–34 with a mean of 6.0 (*n* = 50), which indicates biomagnification along the food chain in every regarded study. The chub samples from 2017 showed a TMF of 16.6, indicating biomagnification within this species [6]. For the diverse fish species sampled in 2020, however, the TMF for PCB 153 was 0.1, indicating trophic dilution. According to Walters et al. [45], this value may indicate that this sample set is not suited to represent biomagnification for this food web.

Similarly, contradictory observations can also be found in the literature. In a comprehensive biomagnification study in a trophic network in a German lake, Kosfeld et al. [46] found a positive log-linear relationship between *C*_Lip_ of HOCs and the TL of fish. In contrast, Dufour et al. [47] did not find a correlation between *C*_Lip_ of HOCs and the TL of Arctic char (*Salvelinus alpinus*) in Lake Geneva, Switzerland.

For the present study, one explanation for the observations described above could be an unbalanced sample set for the multiple fish species collected in 2020, where rather large (old) individuals of mid-range TL species such as chub and ide were caught, but rather small (young) ones for predatory, high-level TL species such as pike (mature length range 25–63 cm [48], here: 30–33 cm). It seems plausible that pike, almost exclusively piscivorous with a body length >17 cm [49–51], might reach a high TL at a young age while having spent too little time to reach the associated explicit accumulation level of HOCs, which can take several years. By relying on a piscivorous diet from a young age, the rapid growth of young pike may lead to a fast accumulation of ^15^N isotopes due to the rapid increase in body mass, hence elevating the TL of young predatory fish [6, 52], which potentially requires a considerably longer time for the corresponding biomagnification of HOCs. Similarly, Borgå et al. [6] also point out that larger organisms that increase their body mass slowly show higher lipid-normalized concentrations of HOCs due to less growth dilution compared to younger, fast-growing individuals. In addition, the range of TL in the chub samples (from 2017) encompassed 1.12 TLs, and the multiple species sampled in 2020 encompassed 1.17 TLs. Hence, both sample sets still represent a relatively narrow TL range, considering that two entire TLs would be desirable for observing significant biomagnification [6]. This narrow TL range might be part of the reason for the lack of a positive correlation between biomagnification of HOCs and TL in the samples from 2020.

McIntyre and Beauchamp [53] point out that biomagnification depends on the accumulation of HOCs attributed to the time spent feeding on a certain TL. If an organism shifts its diet and feeds from a higher TL, the biomagnification rate also increases. However, when a shift in diet from a lower to a higher TL occurs, the higher biomagnification rate takes a certain time to overrule the previous long period of low accumulation from a lower TL. Typically, as an individual fish feeds on a certain TL, it grows in length over time. Once a certain length, corresponding to larger body size, including mouth width, is reached, it has access to bigger prey and, thus, potentially to a higher TL. Hence, we hypothesize that body length can be used as an alternative metric for biomagnification, since it (i) indicates time spent with a certain biomagnification rate and (ii) regulates the access to feeding on higher TL organisms and thus increased biomagnification rates.

**Fig. 2:**
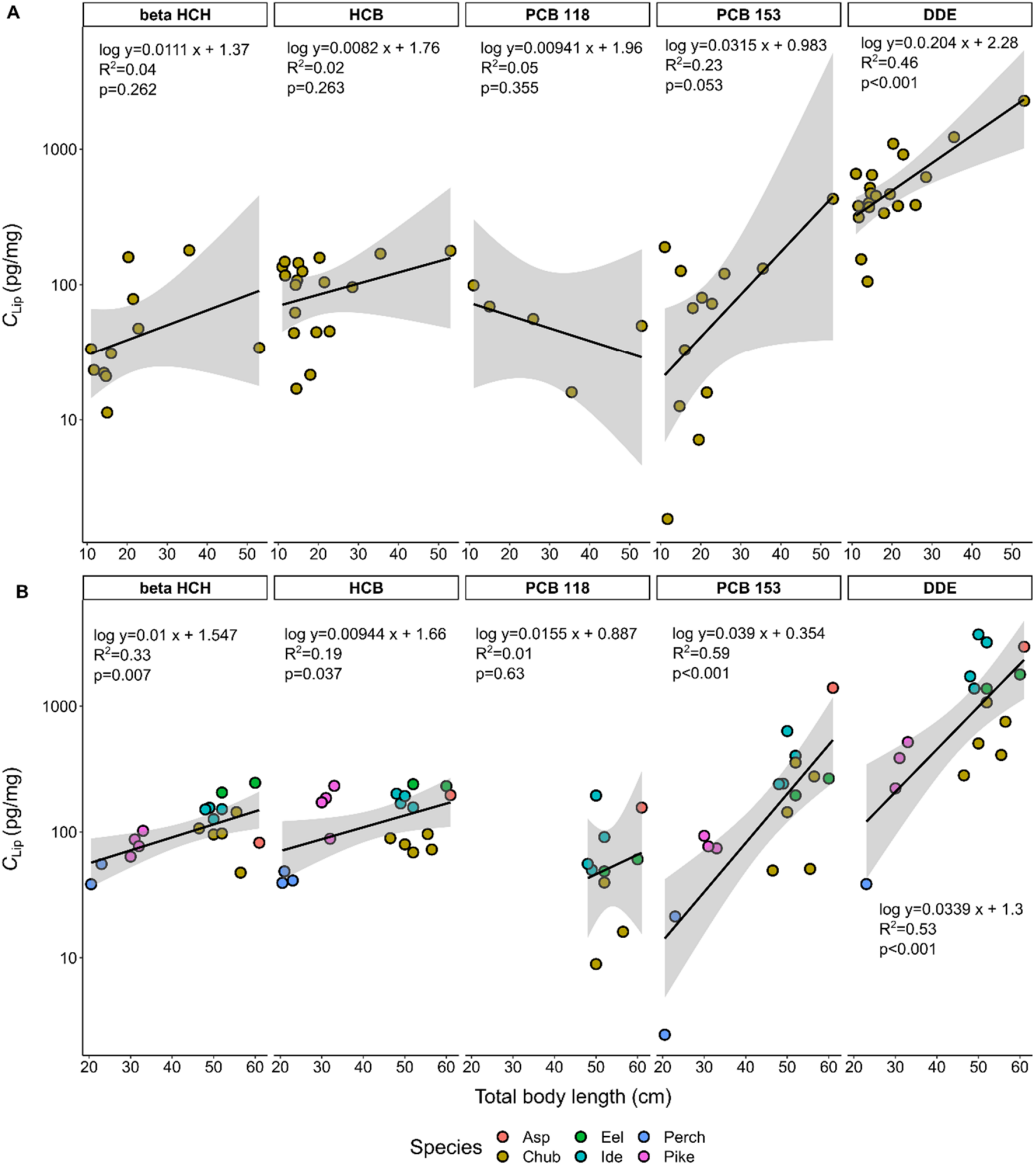
Lipid-normalized concentrations plotted against body length of **A** individuals of European chub sampled in 2017 and **B** diverse fish species sampled in 2020 for the compounds beta HCH, HCB, PCB 118, PCB 153 and DDE. Due to outlier behavior, catfish was excluded from the analysis. A log-linear model is plotted as an equation in each panel and as a black line, along with the grey band illustrating the 95% confidence interval

For European chub from 2017, the body length ranged from 11 to 53 cm. For physiological parameters of individual fish from 2020, see Table 1. In Fig. 2, *C*_Lip_ is plotted against body length for the two sample sets from 2017 (A) and 2020 (B). Despite large uncertainties for some compounds (especially PCB 118), a positive relationship between *C*_Lip_ and body length is observed for both sample sets for four out of five HOCs (except PCB 118) in chub from 2017. *C*_Lip_ of the non-biomagnifying compounds beta HCH and HCB showed a positive (but non-significant) correlation with increasing body length in both data sets. Furthermore, Fig. 2B shows a significant positive relationship for samples from 2020 for beta HCH and HCB and a strong positive relationship between the accumulation of PCB 153 and DDE with increasing body length. Only DDE had a significant relationship with body length for the chub sample set from 2017 (Fig. 2A).

This result provides insights into body length as a relevant indicator for observed biomagnification that may be more robust than age or TL, but further research is needed to make a conclusive statement. As an alternative parameter for body length or TL, the relative weight of a species *W*_rm_ according to Froese [54] could be used, which encompasses the body length and weight of a fish, along with species-specific parameters, allowing to compare the condition of different fish species with each other. *W*_rm_ was calculated and plotted against *C*_Lip_ but showed weaker correlations than body length. Details are described in Additional file 1: Sect. 9 and Figure S10. In addition, individual body length normalized to the corresponding TL was plotted against *C*_Lip_ for the five model HOCs (Additional file 1: Sect. 9 and Figure S11), but did not prove to be a superior estimator for *C*_Lip_ than body length alone, as proposed in Fig. 2.

**Fig. 3:**
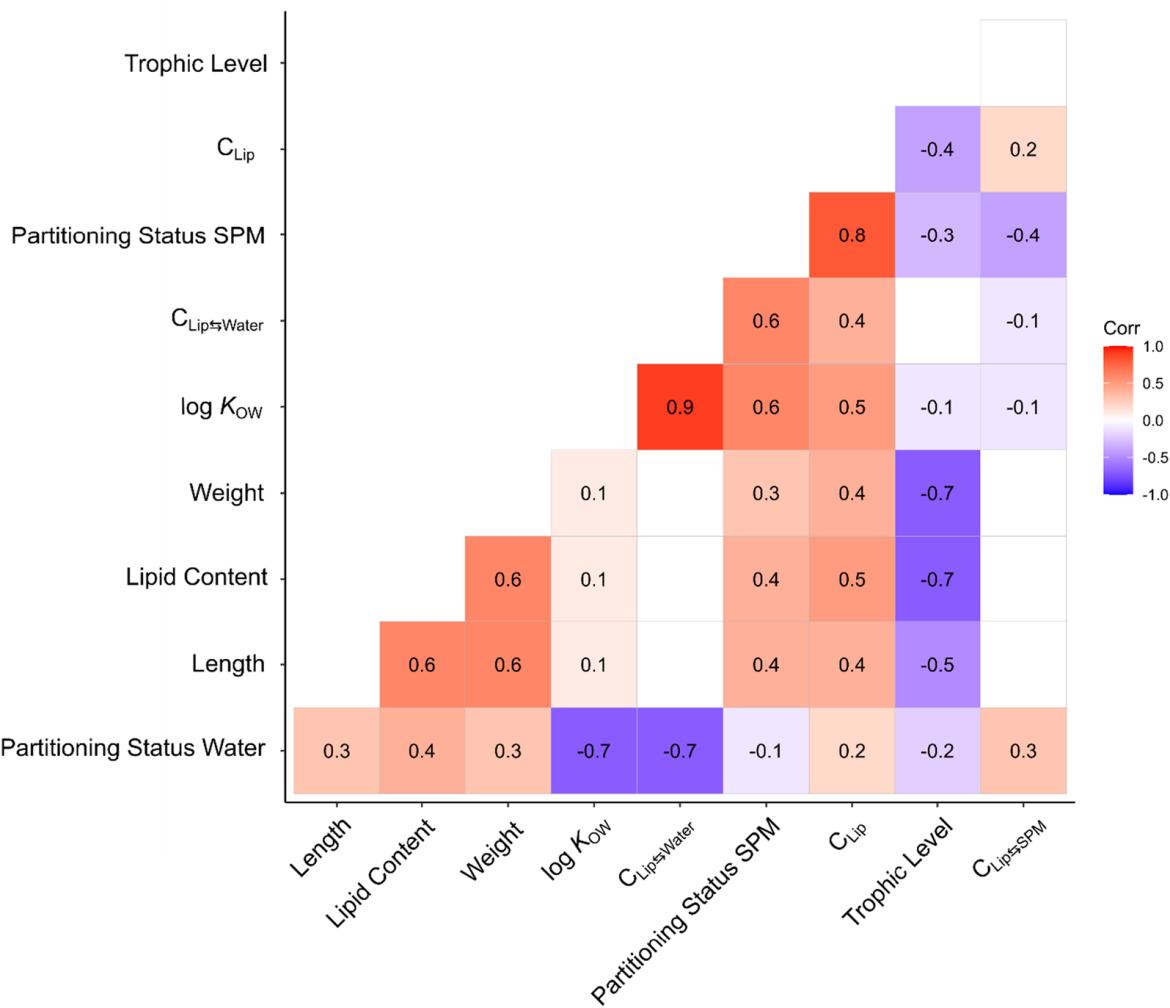
Correlogram for all fish species sampled in 2020 (catfish excluded as an outlier) and the five model HOCs, with Spearman correlation coefficients *ρ* (displayed numbers) for selected parameters. Correlation coefficients ranged from 1 to − 1, indicating a perfect positive correlation between two parameters at a value of 1 and a perfect negative correlation at − 1. A correlation coefficient of 0 indicates no correlation. Non-statistically significant correlations (*p* value > 0.05) are left with an empty field. Concentrations refer to the five model compounds shown in Fig. 2

For a more comprehensive overview of the relations between the diverse parameters of the Mulde 2020 data set, a correlogram is displayed in Fig. 3, showing the statistically significant Spearman correlation coefficients *ρ*. A strong positive correlation was found between log *K*_OW_ and *C*_Lip⇆Water_ (*ρ* = 0.9), whereas the correlation between log *K*_OW_ and *C*_Lip⇆SPM_ (*ρ* = − 0.1) was weakly negative. Furthermore, *C*_Lip_ showed positive correlations with log *K*_OW_ (*ρ* = 0.5), *C*_Lip⇆Water_ (*ρ* = 0.4) and lipid content (*ρ* = 0.5). In addition, lipid content correlates positively with weight (*ρ* = 0.6) and body length (*ρ* = 0.6), but correlates negatively with TL (*ρ* = − 0.7, this was also observed by Dufour et al. [47]). Considering all HOCs, Fig. 3 shows a negative correlation between log *K*_OW_ and the partitioning status for water (*ρ* = − 0.7). In contrast, the partitioning status with SPM correlated positively with log *K*_OW_ (*ρ* = 0.6). TL correlated weakly negatively with the partitioning status with water (*ρ* = − 0.2) and SPM (*ρ* = − 0.3), whereas weak positive correlations were observed for body length (partitioning status water *ρ* = 0.3 and SPM *ρ* = 0.4).

### Partitioning status

When investigating the thermodynamic relationship between water and SPM, *C*_Lip⇆Water_ and *C*_Lip⇆SPM_ (the equilibrium partitioning concentrations in model lipids at thermodynamic equilibrium with the abiotic compartments, water and SPM) of the three major compound groups (PAHs, PCBs and OCPs) can be compared to each other. The lowest ratio of *C*_Lip⇆Water_/*C*_Lip⇆SPM_ was found for beta HCH with 0.01 and the highest for PCB 153 with 0.43, as shown in Additional file 1: Figure S7. Overall, *C*_Lip⇆SPM_ in all cases exceeded *C*_Lip⇆Water_. This observation indicates a higher fugacity in SPM relative to water, resulting in SPM being a source of HOCs relative to the surrounding water and hence is indicative of a resulting mass flow from the SPM into the water.

**Fig. 4:**
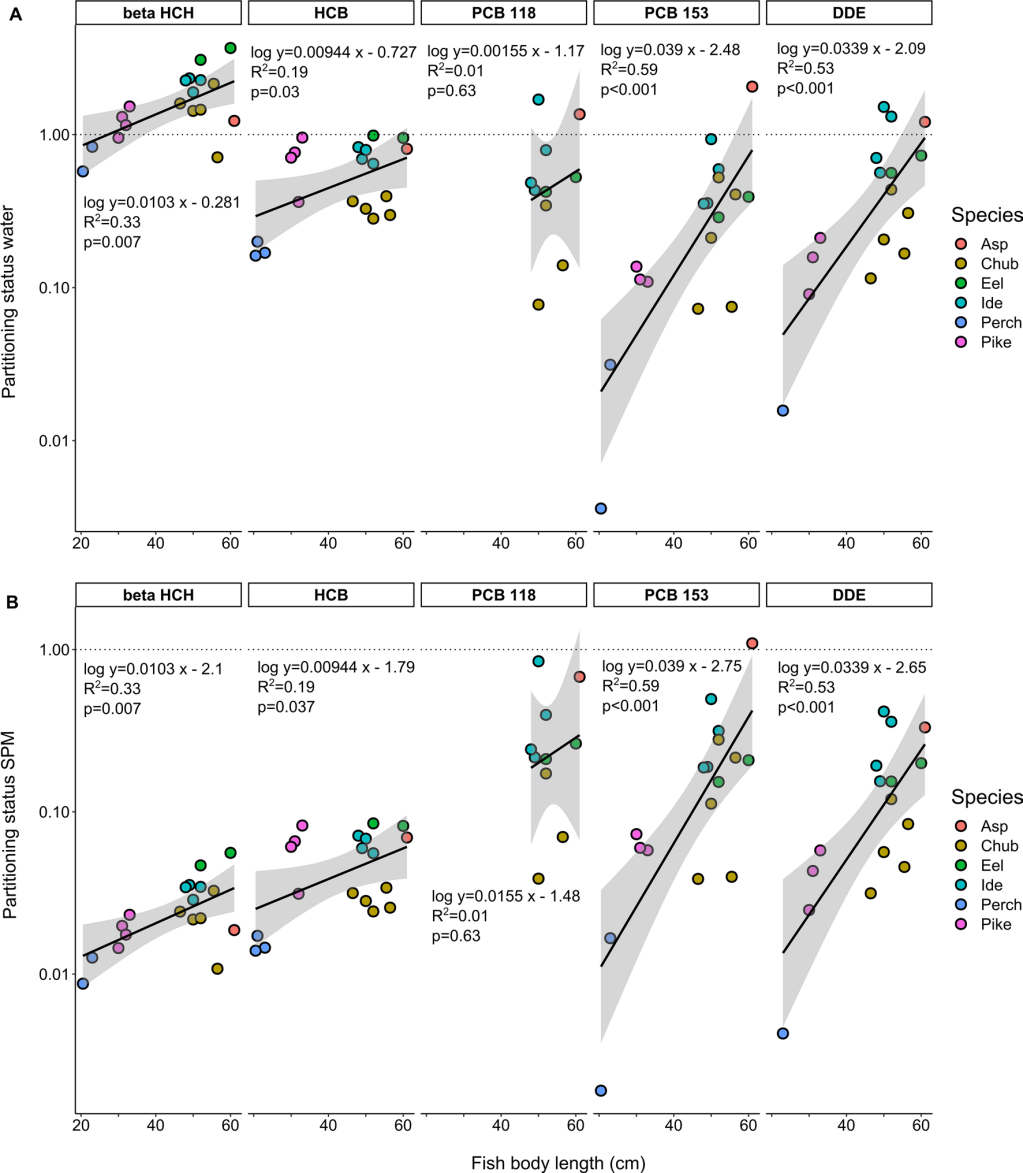
Plot of the mean partitioning status of beta HCH, HCB, PCB 118, PCB 153 and DDE for water (**A**) and SPM (**B**) against the body length of eel, ide, perch, chub, pike and asp sampled in 2020. A log-linear model is plotted as an equation in each panel and as a black line, along with the grey band illustrating the 95% confidence interval. The dotted line marks the thermodynamic equilibrium of lipids with the respective environmental medium. Data points for PCB 118 only encompass quantifiable concentrations in fish between 48 and 61 cm in body length.

Analogously to the discussed thermodynamic relationship above, *C*_Lip_ measured in fish can be set as a ratio against *C*_Lip⇆Water_ or *C*_Lip⇆SPM_ (referred to as partitioning status, Eq. 8). Figure 4 shows that all HOCs had a systematically higher partitioning status between fish and water than between fish and SPM. For comparison, Ghosh et al. [58] demonstrated that surface water, compared to sediment pore water, had a major thermodynamic contribution towards the bioaccumulation of PCBs in largemouth bass in Western Lake Erie. However, this contribution decreased with increasing log *K*_OW_. In general, all compounds in this study showed a positive relationship between increasing partitioning status and increasing body length of fish, as shown for the model compounds in Fig. 2.

As individual compounds were concerned, the partitioning status of beta HCH was > 1 relative to water for all samples (except one individual chub and pike and all individuals of perch) and the highest for eel, followed by other rather lipid-rich species (see Table 1). Similar to beta HCH, the partitioning status for HCB was one order of magnitude higher relative to water than to SPM but ranged between 0.1 and 1 in the case of water. For PCB 118, the partitioning status was mostly below 1, but exceeded 1 for asp and an individual of ide. The steepest slope for the linear model (0.039) was calculated for the partitioning status of PCB 153, where asp had a partitioning status > 1 relative to water and SPM. For DDE, asp and individual ide exceeded the partitioning status of 1 relative to water. The mean ratio ± standard deviation between the partitioning status relative to water and SPM across all species was 84.6 ± 33.9 for beta HCH, 7.3 ± 0.3 for HCB, 24.1 ± 0.9 for PCB 118, 2.3 ± 1.2 for PCB 153 and 3.4 ± 1.3 for DDE.

Pike had the highest TL as derived from the *δ*^15^N data, but exceeded a partitioning status of 1 only for beta HCH when derived from PSDs in water. Since the DEQ for compounds with log *K*_OW_ >5.5 was low, an extrapolation-associated overestimation of *C*_Lip⇆Water_ has to be considered, which could lead to an increasingly uncertain partitioning status with the water phase for those very hydrophobic compounds. Asp exceeded the partitioning status of 1 the most often (beta HCH, PCB 118, 153 and DDE for water and PCB 153 for SPM). The difference in partitioning status between the two top predatory fish (i.e., for DDE, 0.05 and 1.23) in the investigated ecosystem might be due to the large differences in body length and thus age. As discussed in Sect. 3.3, body length may be used as a suitable indicator for age, as it is suggested to represent the individual’s feeding behavior well and give a valid indication of biomagnification. The sampled pike individuals had a relatively short body length (30–33 cm) for their species, suggesting a fast growth rate (including rapid assimilation of lipids) and thus, a high growth dilution of the concentrations of accumulated HOCs. On the other hand, the single asp had a body length of 61 cm, indicating a relatively old age, slow growth and higher biomagnification rate.

### Implications for monitoring efforts

In this study, fish muscle tissue was extracted and analyzed for HOCs, yet other body compartments can accumulate higher amounts of HOCs [55]. This situation can imply uncertainties when findings are extrapolated to entire body burdens. However, the muscle tissue is relevant for human consumption and, therefore, suitable as a reference phase for PSDs in monitoring, whereas the entire body burden may be more suitable for assessing aquatic biomagnification. Since, in this study, the internal exposure concentration was normalized to the tissue’s lipid fraction, the actual body burden of the fish can vary with different lipid contents. Lipid content varied between species (see Table 1), so that, for example, eel upon consumption could deliver a higher contamination load than asp, even though *C*_Lip_ in asp is higher. The uneven distribution of lipids within the fish can also influence the contaminant uptake by the consumer, depending on which parts of the fish are consumed [56, 57].

As discussed in Sect. 3.3, for the present data set, body length appeared to be a more appropriate parameter than TL of the investigated fish for interpreting biomagnification and *C*_Lip_ in different species of the studied small sample sets. Despite not being without challenges regarding its application across different species and age groups, body length has shown potential to be a robust measure to explain the observed biomagnification in a fish. This observation seems plausible due to the close links between body length and mouth width, which may govern access to prey items of different sizes. Furthermore, the measurement of body length does not require the killing of the fish and can easily be done by non-specialists without the need for sophisticated equipment, which makes this parameter a very easily accessible one for risk assessment, also for the general population.

As described in Sect. 3.4, SPM mostly had a conservative character as a proxy for bioaccumulation in the investigated fish species. Only in extreme cases for very hydrophobic PCBs in individual fish of great body length the partitioning status exceeded 1. *C*_Lip⇆Water_ was for all five investigated HOCs closer to the measured *C*_Lip_ than *C*_Lip⇆SPM_. *C*_Lip_ of beta HCH and HCB was for all species within a factor of 5 of *C*_Lip⇆Water_. For more hydrophobic HOCs, the body length of the individual fish had a significant influence on the partitioning status of individuals relative to water as well as to SPM. Those very big fish are of particular interest for human consumption. For those cases, it seems particularly important to establish a proxy of a more conservative character, such as SPM, to be protective of human dietary exposure.

Our acquired sample set of fish is limited in how they represent the complete trophic network of the River Mulde. Hence, additional studies comprising more inclusive sampling are required to confirm that the suggested approach generally works. Yet, water appeared to reflect the partitioning status for fish more realistically. PSDs equilibrated with SPM appeared to be more suitable for a protective estimation of the internal HOC exposure of different fish species, since their more conservative character encompassed even bigger and older fish which are of particular interest for human consumption.

## Supplementary Information

**Additional file 1. **The Supporting Information gives additional information on chemicals used, analytical procedures, lipid extraction, exhaustive extraction of biota tissue, passive sampling of water and SPM, quality assurance and control. Additional data are provided for partition coefficients taken and/or derived from literature.

**Additional file 2: Table S8. **contains the concentrations of analytes in fish (2020) and passive samplers from water and SPM. **Table S9.** lists the coating thicknesses of the coated jars used for each sample and compound. **Table S10.** contains the concentrations of analytes in fish (chub) samples in 2017.

## Data Availability

All data generated or analyzed during this study are included in this published article and its additional files.

## Abbreviations

DDD: Dichlorodiphenyldichloroethane
DDE: Dichlorodiphenyldichloroethylene
DDT: Dichlorodiphenyltrichloroethane
DEQ: Degree of equilibrium
HCB: Hexachlorobenzene
HCH: Hexachlorocyclohexane
HOC: Hydrophobic organic compound
IS: Internal standard
OC: Organic carbon
OCP: Organochlorine pesticide
PAH: Polycyclic aromatic hydrocarbon
PCB: Polychlorinated biphenyl
PRC: Performance reference compound
PSD: Passive sampling device
SPM: Suspended particulate matter
TL: Trophic level

## References

[1] Gobas F (2018). A chemical activity approach to exposure and risk assessment of chemicals: Focus articles are part of a regular series intended to sharpen understanding of current and emerging topics of interest to the scientific community. Environ Toxicol Chem.

[2] Gobas FAPC (1999). Mechanism of biomagnification in fish under laboratory and field conditions. Environ Sci Technol.

[3] Macdonald R, Mackay D, Hickie B (2002). Contaminant amplification in the environment. Environ Sci Technol.

[4] Qiao P, Gobas FA, Farrell AP (2000). Relative contributions of aqueous and dietary uptake of hydrophobic chemicals to the body burden in juvenile rainbow trout. Arch Environ Contam Toxicol.

[5] Kidd KA (1998). Bioaccumulation of organochlorines through a remote freshwater food web in the Canadian Arctic. Environ Pollut.

[6] Borgå K (2012). Trophic magnification factors: considerations of ecology, ecosystems, and study design. Integr Environ Assess Manag.

[7] Walters DM (2008). Influence of Trophic Position and Spatial Location on Polychlorinated Biphenyl (PCB) Bioaccumulation in a Stream Food Web. Environ Sci Technol.

[8] Jardine TD, Kidd KA, Fisk AT (2006). Applications, Considerations, and Sources of Uncertainty When Using Stable Isotope Analysis in Ecotoxicology. Environ Sci Technol.

[9] Cabana G, Rasmussen JB (1994). Modelling food chain structure and contaminant bioaccumulation using stable nitrogen isotopes. Nature.

[10] RCC R.C.C.f.E.a.S. Environment and Society Portal. 2022. https://www.environmentandsociety.org/exhibitions/neva-and-danube-rivers/fish-consumption.

[11] FIZ F. Fischwirtschaft Daten und Fakten. 2020: Hamburg.

[12] Counihan TD (2018). Can data from disparate long-term fish monitoring programs be used to increase our understanding of regional and continental trends in large river assemblages?. PLoS ONE.

[13] Jennings S, Rice J (2011). Towards an ecosystem approach to fisheries in Europe: a perspective on existing progress and future directions. Fish Fish.

[14] Jahnke A (2014). Equilibrium sampling to determine the thermodynamic potential for bioaccumulation of persistent organic pollutants from sediment. Environ Sci Technol.

[15] Schäfer S (2015). Equilibrium sampling of polychlorinated biphenyls in River Elbe sediments – Linking bioaccumulation in fish to sediment contamination. Chemosphere.

[16] Rojo-Nieto E, Perales JA (2015). Estimating baseline toxicity of PAHs from marine chronically polluted sediments and bioaccumulation in target organs of fish hypothetically exposed to them: a new tool in risk assessment. Environ Sci Process Impacts.

[17] Wernicke T (2022). Equilibrium sampling of suspended particulate matter as a universal proxy for fish and mussel monitoring. Ecotoxicol Environ Saf.

[18] Niu L (2021). Suspended Particulate Matter-A Source or sink for chemical mixtures of organic micropollutants in a small river under baseflow conditions?. Environ Sci Technol.

[19] Smedes F (2020). Unraveling the relationship between the concentrations of hydrophobic organic contaminants in freshwater fish of different trophic levels and water using passive sampling. Environ Sci Technol.

[20] Joyce AS (2016). Evaluating the relationship between equilibrium passive sampler uptake and aquatic organism bioaccumulation. Environ Sci Technol.

[21] Schmidt SN, Burgess RM (2020). Evaluating polymeric sampling as a tool for predicting the bioaccumulation of polychlorinated biphenyls by fish and shellfish. Environ Sci Technol.

[22] Paschke A (2006). Comparative application of solid-phase microextraction fibre assemblies and semi-permeable membrane devices as passive air samplers for semi-volatile chlorinated organic compounds. A case study on the landfill "Grube Antonie" in Bitterfeld. Germany. Environ Pollution.

[23] LHW (2013) Sachstandsbericht zur Schadstoffbelastung der Oberflächengewässer in Sachsen-Anhalt und zur Identifizierung der Ursachen und Quellen. LHW Sachsen-Anhalt, Gewässerkundlicher Landesdienst: Magdeburg.

[24] Fliedner A (2016). Current levels and trends of selected EU Water Framework Directive priority substances in freshwater fish from the German environmental specimen bank. Environ Pollut.

[25] Reichenberg F (2008). Determining the chemical activity of hydrophobic organic compounds in soil using polymer coated vials. Chem Cent J.

[26] Vrana B (2019). Chasing equilibrium passive sampling of hydrophobic organic compounds in water. Sci Total Environ.

[27] Booij K, Smedes F (2010). An improved method for estimating in situ sampling rates of nonpolar passive samplers. Environ Sci Technol.

[28] Smedes F, Booji K (2012). Guidelines for passive sampling of hydrophobic contaminants in water using silicone rubber samplers. ICES Techn Mar Environ Sci.

[29] Muz M, Rojo-Nieto E, Jahnke A (2021). Removing disturbing matrix constituents from biota extracts from total extraction and silicone-based passive sampling. Environ Toxicol Chem.

[30] Ricking MK. Martin; Heiniger, Peter; Körner, Andrea, Richtlinie zur Probenahme und Probenbearbeitung Schwebstoffe V 4.0.3. 2017, Umweltprobenbank des Bundes: Freie Universität Berlin, Fachbereich Geowissenschaften, Arbeitsbereich Hydrogeologie.

[31] Maenpaa K (2011). Equilibrium sampling of persistent and bioaccumulative compounds in soil and sediment: comparison of two approaches to determine equilibrium partitioning concentrations in lipids. Environ Sci Technol.

[32] Jahnke A, Mayer P, McLachlan MS (2012). Sensitive equilibrium sampling to study polychlorinated biphenyl disposition in Baltic Sea sediment. Environ Sci Technol.

[33] Mayer P (2003). Equilibrium sampling devices. Environ Sci Technol.

[34] Schulz-Zunkel C (2022). Effective restoration measures in river-floodplain ecosystems: Lessons learned from the ‘Wilde Mulde’ project. Int Rev Hydrobiol.

[35] Jensen S (2003). A quantitative lipid extraction method for residue analysis of fish involving nonhalogenated solvents. J Agric Food Chem.

[36] R Core Team (2020) R: A Language and Environment for Statistical Computing. R Foundation for Statistical Computing: Vienna, Austria.

[37] Smedes F (2019). SSP silicone-, lipid- and SPMD-water partition coefficients of seventy hydrophobic organic contaminants and evaluation of the water concentration calculator for SPMD. Chemosphere.

[38] Ucar IP (2018). Edzer; Azcorra, Arturo, Measurement Errors in R. R J.

[39] Smedes F (2017). Partitioning of hydrophobic organic contaminants between polymer and lipids for two silicones and low density polyethylene. Chemosphere.

[40] Gilbert D (2016). Polymers as reference partitioning phase: polymer calibration for an analytically operational approach to quantify multimedia phase partitioning. Anal Chem.

[41] Meador JP et al (1995) Bioaccumulation of Polycyclic Aromatic Hydrocarbons by Marine Organisms, In: Ware GW. Reviews of environmental contamination and toxicology. Springer, New York. p. 79–165. 10.1007/978-1-4612-2542-3_4 7501868

[42] Deutsch KA et al (2014) Common Implementation Strategy For The Water Framework Directive (2000/60/EC). Guidance Document No. 32 on biota monitoring (the implementation of eqsbiota) under the water framework directive. CTIT technical reports series.

[43] Post DM (2002). Using stable isotopes to estimate trophic position: models, methods and assumptions. Ecology.

[44] Kelly BC (2007). Food web-specific biomagnification of persistent organic pollutants. Science.

[45] Walters DM (2016). Trophic magnification of organic chemicals: a global synthesis. Environ Sci Technol.

[46] Kosfeld V (2021). Food web on ice: a pragmatic approach to investigate the trophic magnification of chemicals of concern. Environ Sci Eur.

[47] Dufour E (2001). Assessment of the contaminant concentration variability among Lake Geneva Arctic char using stable isotopic composition (δ15N and δ13C). Environ Toxicol.

[48] Froese R, Pauly D. FishBase. 2019. www.fishbase.org.

[49] Diana JS (1979). The feeding pattern and daily ration of a top carnivore, the northern pike (Esox lucius). Can J Zool.

[50] Mann RHK (1982). The annual food consumption and prey Preferences of Pike (Esox lucius) in the River Frome. Dorset J Anim Ecol.

[51] Skov C (2011). Dispersal, growth, and diet of stocked and wild northern pike fry in a shallow natural lake, with implications for the management of stocking programs. North Am J Fish Manag.

[52] Hesslein RH, Hallard KA, Ramlal PS (1993). Replacement of Sulfur, Carbon, and Nitrogen in Tissue of Growing Broad Whitefish (Coregonus nasus) in Response to a Change in Diet Traced by d34S, d13C, and d15N. Can J Fish Aquat Sci.

[53] McIntyre JK, Beauchamp DA (2007). Age and trophic position dominate bioaccumulation of mercury and organochlorines in the food web of Lake Washington. Sci Total Environ.

[54] Froese R (2006). Cube law, condition factor and weight–length relationships: history, meta-analysis and recommendations. J Appl Ichthyol.

[55] Endo S, Brown TN, Goss KU (2013). General model for estimating partition coefficients to organisms and their tissues using the biological compositions and polyparameter linear free energy relationships. Environ Sci Technol.

[56] Toussaint C (2005). Description of the heterogeneity of lipid distribution in the flesh of brown trout (Salmo trutta) by MR imaging. Aquaculture.

[57] Nanton DA (2007). Muscle lipid storage pattern, composition, and adipocyte distribution in different parts of Atlantic salmon (Salmo salar) fed fish oil and vegetable oil. Aquaculture.

[58] Ghosh U, Bokare M, Gobas FAPC (2021). Deconvoluting thermodynamics from biology in the aquatic food web model. Environ Toxicol Chem.

