# Supplementary material for: Exploring the partitioning of hydrophobic organic compounds between water, suspended particulate matter and diverse fish species in a German river ecosystem
Source: Environ Sci Eur. 2022 Aug 5;34(1):66. doi: 10.1186/s12302-022-00644-w (PMC9355927; doi:10.1186/s12302-022-00644-w)
Supplement: Supplementary file 1 — Additional file 1. The Supporting Information gives additional information on chemicals used, analytical procedures, lipid extraction, exhaustive extraction of biota tissue, passive sampling of water and SPM, quality assurance and control. Additional data are provided for partition coefficients taken and/or derived from literature. [file 12302_2022_644_MOESM1_ESM.docx]

**Supporting Information**

**Exploring the Partitioning of Hydrophobic Organic Compounds between Water, Particulate Matter and Diverse Fish Species in a German River Ecosystem**

Theo Wernicke, Elisa Rojo-Nieto, Albrecht Paschke, Claudia Nogueira Tavares, Mario Brauns, Annika Jahnke

Table of Content

[List of Figures S2](#_Toc100239997)

[List of Tables S3](#_Toc100239998)

[1. Field Sampling Site S4](#_Toc100239999)

[2. Chemicals S5](#_Toc100240000)

[3. Chemical Analysis S9](#_Toc100240001)

[4. Quality Assurance and Quality Control S10](#_Toc100240002)

[5. Passive Sampling of Water S14](#_Toc100240003)

[6. Passive Sampling of SPM S15](#_Toc100240004)

[7. Stable Isotope Analysis S15](#_Toc100240005)

[8. Partitioning between Water and SPM S18](#_Toc100240006)

[9. Additional Results for Fish S23](#_Toc100240007)

[References S28](#_Toc100240008)

# List of Figures

[Figure S1: Map of sampling sites (black triangles) along the River Mulde (orange path) near Dessau, 51.731550, 12.295652. Base map adapted from openstreetmap.org. S4](#_Toc108705326)

[Figure S2: Example of a nls-model for the fraction of retained PRCs as a function of log (K_pw_M^0.47^) fitted to data from SSP sampler Nr. 2. S14](#_Toc108705327)

[Figure S3: Plot of δ^15^N against δ^13^C for chub (2017) (A) and fish species from 2020 (B). S17](#_Toc108705328)

[Figure S4 Mean C_Lip⇆SPM_ against mean C_Lip⇆Water_ for PAHs. The diagonal line symbolizes the 1:1 ratio. Error bars represent the standard deviation of the mean. S19](#_Toc108705329)

[Figure S5: Mean C_Lip⇆SPM_ against mean C_Lip⇆Water_ for PCBs. The diagonal line symbolizes the 1:1 ratio. Error bars represent the standard deviation of the mean. S20](#_Toc108705330)

[Figure S6: Mean C_Lip⇆SPM_ against mean C_Lip⇆Water_ for OCPs. The diagonal line symbolizes the 1:1 ratio. Error bars represent the standard deviation of the mean. S21](#_Toc108705331)

[Figure S7: Ratio between C_Lip⇆Water_ and C_Lip⇆SPM_ plotted against the log K_OW_ of each individual chemical. The dotted line marks the ratio = 1. A linear model is given as an equation and as a black line, along with the grey band illustrating the 95% confidence interval. S22](#_Toc108705332)

[Figure S8: Lipid-normalized concentrations in the investigated fish samples plotted against trophic level of (A) individuals of European chub sampled in 2017 and (B) all fish species (catfish was excluded) sampled in 2020 for the model compounds beta HCH, HCB, PCB 118, PCB 153 and DDE spanning a wide range of hydrophobicities. A log-linear model is plotted as an equation in each panel and as a black line, along with the grey band illustrating the 95% confidence intervals. S23](#_Toc108705333)

[Figure S9: Plot of the mean partitioning status of Acenaphthylene, Anthracene and Benzo(ghi)perylene for water (A) and SPM (B) against the body length of eel, ide, perch, chub, pike and asp sampled in 2020. A log-linear model is plotted as an equation in each panel and as a black line, along with the grey band illustrating the 95% confidence interval. S24](#_Toc108705334)

[Figure S10: Lipid-normalized concentrations plotted against relative weight W_rm_ according to Froese [8] of (A) individuals of European chub sampled in 2017 and (B) all fish species (catfish was exclude) sampled in 2020 for the compounds beta HCH, HCB, PCB 118, PCB 153 and DDE. A log-linear model is plotted as an equation in each panel and as a black line, along with the grey band illustrating the 95% confidence interval. S26](#_Toc108705335)

[Figure S11: Lipid-normalized concentrations plotted against TL-normalized body length of (A) individuals of European chub sampled in 2017 and (B) all fish species (catfish was excluded from the analysis) sampled in 2020 for the compounds beta HCH, HCB, PCB 118, PCB 153 and DDE. A log-linear model is plotted as an equation at the top of each plot and as a black line, along with the grey band illustrating the 95% confidence interval. S27](#_Toc108705336)

# List of Tables

[Table S1: List of target compounds and isotope-labeled internal standards (IS) analyzed in GC/MS dMRM mode. One ion transition (m/z) was used as quantifier and at least one m/z was used as qualifier for the respective compounds. A second qualifier m/z was added in cases of insufficient primary qualifier intensity. S5](#_Toc108698762)

[Table S2: Analyzed compounds with corresponding compound groups, log K_OW_, K_Lip:DC_ (for the polymer coated on jars), K_Lip:SSP_ (for the polymer used in water sampling), log K_DC:water_ and the MQL values of the extraction method of biota tissue (JenII), coated jars (CJ) and PSDs in water (PSD). S11](#_Toc108698763)

[Table S3: Modelling results for the four SSP samplers deployed in water, with the estimates for B, the corresponding standard error and the estimates for R_S_ for DDE as an example compound. S14](#_Toc108698764)

[Table S4: Means and standard deviations of stable isotope data for carbon and nitrogen for all invertebrate species investigated in 2017. S16](#_Toc108698765)

[Table S5: Means and standard deviations of stable isotope data for carbon and nitrogen for all invertebrate species investigated in 2020. S16](#_Toc108698766)

[Table S6: List with mean and standard deviations (sd) equilibrium model lipid concentrations derived from PSD sampling of water and SPM. S18](#_Toc108698767)

[Table S7: Species specific model parameters taken from FishBase. S25](#_Toc108698768)

# Field Sampling Site


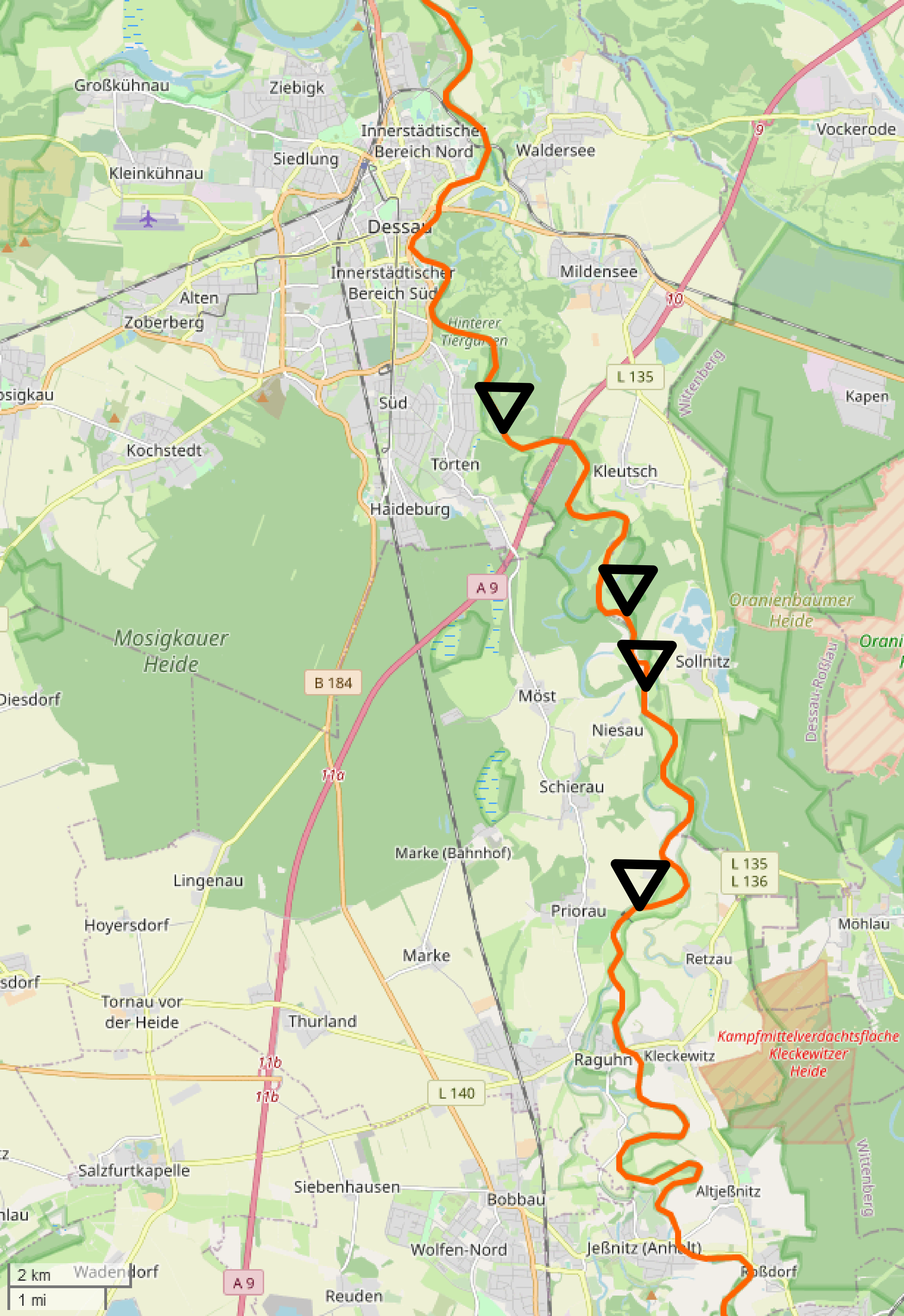


Figure S1: Map of sampling sites (black triangles) along the River Mulde (orange path) near Dessau, 51.731550, 12.295652. Base map adapted from openstreetmap.org.

# Chemicals

Acetone and acetonitrile were purchased from Th. Geyer (Germany); ethyl acetate, pentane, methanol and hexane were purchased from Sigma-Aldrich (Germany). All solvents had GC/MS grade quality. Triolein was purchased from Sigma-Aldrich (Germany). Analytical standards used for data quantification consisted of eight polychlorinated biphenyls (PCBs), 14 polycyclic aromatic hydrocarbons (PAHs) and hexachlorobenzene (HCB), as well as alpha, beta, gamma and delta hexachlorocyclohexane (HCH), dichlorodiphenyltrichloroethane (p,p`-DDT) and its metabolites dichlorodiphenyldichloroethane (p,p`-DDD) and Dichlorodiphenyldichloroethylene (p,p`-DDE). Besides those compounds, we also measured additional pollutants (Table S1) which are not discussed in the main manuscript due to lack of partition coefficient data. Stable isotope-labeled internal standards (IS) used in this study were: Naphthalene d8, Fluorene d16, Pyrene d10, Chrysene d12, Benzo(a)pyrene d12, Dibenz(a,h)anthacene d14, Acenaphthylene d8, Anthracene d10, Phenanthrene d10, Pyrene d10, ^13^C_6_ PCB 28, ^13^C_6_ PCB 52, ^13^C_6_ PCB 101, ^13^C_6_ PCB 118, ^13^C_6_ PCB 153, ^13^C_6_ PCB 138, ^13^C_6_ PCB 180, 4,4 DDT d8, ^13^C_3_ HCB, Tonalide d3, mixed in an ethyl acetate stock solution (1000 pg/µL). Performance Reference Compounds (PRCs) used in water PSDs were PCB 10, 30 and 104, Acenaphthylene d8, Anthracene d10 and Fluoranthene d10.

Table S1: List of target compounds and isotope-labeled internal standards (IS) analyzed in GC/MS dMRM mode. One ion transition (m/z) was used as quantifier and at least one m/z was used as qualifier for the respective compounds. A second qualifier m/z was added in cases of insufficient primary qualifier intensity.

| **Compound** | **associated IS** | **Type** | **CAS Nr.** | **Supplier** | **Retention Time (minutes)** | **Quantifier Transition (*m/z*)** | **Quantifier Collision Energy (eV)** | **Qualifier Transition 1 (*m/z*)** | **Qualifier Transition 1 Collision Energy (eV)** | **Qualifier Transition 2 (*m/z*)** | **Qualifier Transition 2 Collision Energy (eV)** |
| --- | --- | --- | --- | --- | --- | --- | --- | --- | --- | --- | --- |
| 4,4'-DDT d8 | - | IS | 93952-18-2 | Sigma Aldrich | 29.3 | 242.9 -> 173.2 | 30 | 242.9 -> 205.9 | 25 | - | - |
| Benzo(a)pyrene d12 | - | IS | 63466-71-7 | Dr. Ehrenstorfer | 42.2 | 264.0 -> 260.3 | 40 | 132.0 -> 118.0 | 15 | - | - |
| Chrysene d12 | - | IS | 1719-03-5 | Dr. Ehrenstorfer | 31.8 | 240.0 -> 236.2 | 40 | 240.0 -> 212.3 | 35 | 119.9 -> 105.9 | 15 |
| Dibenz(ah)anthracene d14 | - | IS | 13250-98-1 | Dr. Ehrenstorfer | 49.4 | 292.0 -> 288.3 | 40 | 146.0 -> 132.0 | 15 | - | - |
| Fluorene d16 | - | IS | 81103-79-9 | Dr. Ehrenstorfer | 12.0 | 176.0 -> 174.1 | 25 | 145.9 -> 94.2 | 25 | - | - |
| Hexachlorobenzene ^13^C_3_ | - | IS | 35065-28-2 | Dr. Ehrenstorfer | 14.6 | 254.8 -> 219.9 | 15 | 291.8 -> 222.0 | 20 | 291.8 -> 256.9 | 20 |
| PCB 101 ^13^C_6_ | - | IS | 103130-39-4 | Campro Scientific | 24.2 | 265.9 -> 196.1 | 40 | 337.8 -> 268.0 | 30 | - | - |
| PCB 118 ^13^C_6_ | - | IS | - | Campro Scientific | 27.1 | 337.8 -> 268.1 | 35 | 265.9 -> 195.9 | 35 | - | - |
| PCB 138 ^13^C_6_ | - | IS | 208263-66-5 | Campro Scientific | 29.3 | 301.8 -> 230.0 | 35 | 371.8 -> 337.1 | 35 | - | - |
| PCB 153 ^13^C_6_ | - | IS | 185376-58-3 | Campro Scientific | 28.5 | 371.8 -> 302.0 | 35 | 371.8 -> 337.1 | 25 | 301.8 -> 232.1 | 40 |
| PCB 180 ^13^C_6_ | - | IS | 35065-29-3 | Campro Scientific | 32.8 | 405.8 -> 335.9 | 35 | 405.8 -> 371.1 | 25 | 335.8 -> 265.9 | 35 |
| PCB 28 ^13^C_6_ | - | IS | 7012-37-5 | Campro Scientific | 18.6 | 267.9 -> 198.1 | 30 | 198.0 -> 163.2 | 30 | - | - |
| PCB 52 ^13^C_6_ | - | IS | 35693-99-3 | Campro Scientific | 20.3 | 231.9 -> 162.2 | 40 | 231.9 -> 197.1 | 25 | 303.8 -> 234.0 | 30 |
| Pyrene d10 | - | IS | 1718-52-1 | Dr. Ehrenstorfer | 24.3 | 212.0 -> 208.2 | 35 | 212.0 -> 210.2 | 45 | - | - |
| Tonalide d3 | - | IS | - | Neochema | 18.0 | 261.0 -> 246.0 | 15 | 261.0 -> 190.0 | 15 | 261.0 -> 160.0 | 25 |
| Acenaphthylene d8 | Fluorene d16 | Target | - | Sigma Aldrich | 9.5 | 160.0 -> 132.0 | 30 | 160.0 -> 80.0 | 40 | - | - |
| Anthracene d10 | Fluorene d16 | Target | - | Sigma Aldrich | 16.8 | 188.0 -> 183.9 | 40 | 188.0 -> 183.9 | 40 | - | - |
| Fluoranthene d10 | Pyrene d10 | Target | - | Sigma Aldrich | 16.9 | 212.0 -> 208.0 | 40 | - | - | - | - |
| PCB 10 | Hexachlorobenzene ^13^C_3_ | Target | - | Sigma Aldrich | 12.7 | 221.9 -> 151.9 | 30 | 221.9 -> 185.9 | 20 | - | - |
| PCB 30 | PCB 28 ^13^C_6_ | Target | - | Sigma Aldrich | 15.7 | 255.8 -> 185.9 | 10 | 186.0 -> 150.0 | 20 | - | - |
| PCB 104 | PCB 101 ^13^C_6_ | Target | - | Sigma Aldrich | 20.9 | 253.8 -> 183.9 | 30 | 325.8 -> 253.9 | 20 | - | - |
| 4,4'-DDD | 4,4'-DDT d8 | Target | 72-54-8 | HPC Standards | 27.5 | 234.9 -> 165.1 | 25 | 234.9 -> 198.9 | 20 | - | - |
| 4,4'-DDT | 4,4'-DDT d8 | Target | 50-29-3 | HPC Standards | 29.3 | 245.8 -> 176.1 | 35 | 317.8 -> 246.0 | 25 | - | - |
| 4,4'-DDE | 4,4'-DDT d8 | Target | 72-55-9 | HPC Standards | 25.6 | 234.9 -> 165.1 | 30 | 234.9 -> 199.0 | 20 | - | - |
| Acenaphtylene | Fluorene d16 | Target | 208-96-8 | Dr. Ehrenstorfer | 9.5 | 151.9 -> 151.1 | 25 | 151.9 -> 126.0 | 30 | 151.9 -> 102.1 | 35 |
| αHexachlorocyclohexane | Hexachlorobenzene ^13^C_3_ | Target | 319-84-6 | HPC Standards | 14.4 | 218.8 -> 182.9 | 5 | 182.8 -> 147.0 | 15 | - | - |
| Anthracene | Phenanthrene d10 | Target | 120-12-7 | Dr. Ehrenstorfer | 16.9 | 177.9 -> 152.1 | 30 | 177.9 -> 176.1 | 30 | 151.9 -> 151.0 | 20 |
| Benzo(a)pyrene | Benzo(a)pyrene d12 | Target | 50-32-8 | Dr. Ehrenstorfer | 42.3 | 251.9 -> 250.1 | 40 | 126.0 -> 112.9 | 15 | - | - |
| Benzo(b)fluoranthene | Benzo(a)pyrene d12 | Target | 205-99-2 | Dr. Ehrenstorfer | 39.8 | 251.9 -> 250.1 | 40 | 251.9 -> 226.1 | 35 | 126.0 -> 113.1 | 15 |
| Benzo(ghi)perylene | Dibenz(ah)anthracene d14 | Target | 191-24-2 | Dr. Ehrenstorfer | 50.4 | 276.0 -> 274.0 | 50 | 137.0 -> 124.2 | 15 | 137.0 -> 136.2 | 15 |
| Benzo(k)fluoranthene | Benzo(a)pyrene d12 | Target | 207-08-9 | Dr. Ehrenstorfer | 40.0 | 251.9 -> 250.1 | 40 | 251.9 -> 226.1 | 35 | 126.0 -> 113.1 | 15 |
| Benzo(a)anthracene | Chrysene d12 | Target | 56-55-3 | Sigma Aldrich | 31.7 | 228.0 -> 226.1 | 35 | 228.0 -> 202.1 | 35 | 114.1 -> 101.1 | 15 |
| βHexachlorocyclohexane | Hexachlorobenzene ^13^C_3_ | Target | 319-85-7 | HPC Standards | 15.9 | 182.8 -> 146.8 | 20 | 182.8 -> 110.9 | 40 | - | - |
| Chrysene | Chrysene d12 | Target | 218-01-9 | Dr. Ehrenstorfer | 31.9 | 228.0 -> 226.1 | 35 | 228.0 -> 202.1 | 35 | 113.1 -> 100.1 | 15 |
| δHexachlorocyclohexane | Hexachlorobenzene ^13^C_3_ | Target | 319-86-8 | HPC Standards | 17.3 | 182.8 -> 147.0 | 15 | 218.8 -> 182.9 | 5 | - | - |
| Dibenz(ah)anthracene | Dibenz(ah)anthracene d14 | Target | 53-70-3 | Dr. Ehrenstorfer | 49.5 | 277.9 -> 276.0 | 40 | 277.9 -> 252.2 | 35 | 137.9 -> 125.0 | 15 |
| Fluoranthene | Pyrene d10 | Target | 206-44-0 | Dr. Ehrenstorfer | 24.3 | 201.9 -> 200.1 | 40 | 201.9 -> 176.0 | 35 | 201.9 -> 152.1 | 35 |
| Fluorene | Fluorene d16 | Target | 86-73-7 | Dr. Ehrenstorfer | 12.1 | 165.9 -> 165.1 | 25 | 165.9 -> 139.0 | 50 | 165.9 -> 115.0 | 50 |
| γHexachlorocyclohexane (Lindane) | Hexachlorobenzene ^13^C_3_ | Target | 58-89-9 | HPC Standards | 15.9 | 180.8 -> 144.9 | 15 | 180.8 -> 108.9 | 35 | 218.8 -> 183.0 | 10 |
| HCB | Hexachlorobenzene ^13^C_3_ | Target | 118-74-1 | HPC Standards | 14.6 | 283.8 -> 213.9 | 35 | 283.9 -> 248.9 | 20 | - | - |
| Indeno(123-cd)pyrene | Dibenz(ah)anthracene d14 | Target | 193-39-5 | Dr. Ehrenstorfer | 49.3 | 275.9 -> 274.1 | 45 | 275.9 -> 249.8 | 45 | 137.9 -> 125.0 | 15 |
| ISO E Super | Hexachlorobenzene ^13^C_3_ | Target | 544-57-2 | TRC | 14.1 | 234.1 -> 191.0 | 15 | 191.0 -> 118.9 | 20 | - | - |
| PCB 101 | PCB 101 ^13^C_6_ | Target | 37680-73-2 | Promochem | 24.3 | 253.8 -> 184.0 | 40 | 325.7 -> 255.9 | 35 | - | - |
| PCB 118 | PCB 118 ^13^C_6_ | Target | 31508-00-6 | AccuStandard | 27.1 | 325.8 -> 256.0 | 35 | 325.8 -> 254.0 | 35 | 253.8 -> 184.1 | 40 |
| PCB 138 | PCB 138 ^13^C_6_ | Target | 35065-28-2 | Dr. Ehrenstorfer | 29.3 | 359.7 -> 289.9 | 35 | 359.7 -> 287.9 | 35 | - | - |
| PCB 153 | PCB 153 ^13^C_6_ | Target | 35065-27-1 | Promochem | 28.1 | 359.7 -> 289.9 | 35 | 289.8 -> 255.0 | 40 | 289.8 -> 219.9 | 40 |
| PCB 180 | PCB 180 ^13^C_6_ | Target | 35065-29-3 | Promochem | 32.8 | 393.9 -> 323.9 | 35 | 393.9 -> 358.9 | 20 | 323.7 -> 253.4 | 40 |
| PCB 209 | PCB 180 ^13^C_6_ | Target | 2051-24-3 | Dr. Ehrenstorfer | 42.5 | 497.6 -> 427.7 | 35 | 427.6 -> 357.8 | 40 | - | - |
| PCB 28 | PCB 28 ^13^C_6_ | Target | 7012-37-5 | Dr. Ehrenstorfer | 18.6 | 255.8 -> 186.1 | 30 | 185.9 -> 151.1 | 25 | - | - |
| PCB 52 | PCB 52 ^13^C_6_ | Target | 35693-99-3 | Promochem | 20.3 | 291.8 -> 222.0 | 30 | 291.8 -> 256.7 | 15 | - | - |
| Phenanthrene | Phenanthrene d10 | Target | 85-01-8 | Dr. Ehrenstorfer | 16.6 | 177.9 -> 152.1 | 30 | 177.9 -> 176.1 | 30 | 151.9 -> 151.0 | 20 |
| Pyrene | Pyrene d10 | Target | 129-00-0 | Dr. Ehrenstorfer | 23.2 | 201.9 -> 200.1 | 45 | 201.9 -> 151.1 | 50 | - | - |
| Tonalide | Tonalide d3 | Target | 21145-77-7 | LGC | 18.0 | 243.0 -> 187.2 | 5 | 243.0 -> 201.2 | 10 | 243.0 -> 159.2 | 15 |

# Chemical Analysis

Matrix-method-matched calibrations were prepared for each extraction method, with calibration levels for 0.1, 0.5, 1, 10, 20, 50, 100, 200, 500 and 1000 pg/µL. The IS concentration was adjusted to 200 pg/µL for calibration levels, blanks and samples.

All analyses of hydrophobic organic compounds (HOCs) were done on a 7890A GC System coupled to a 7000 GC/MS TripleQuad (Agilent Technolgies, USA). Mass transitions of precursor/product ions were recorded in dynamic multiple reaction monitoring (dMRM) mode that combines excellent specificity with high sensitivity.

For separation of the chemicals of all extracts, an Agilent 122-5532G column (dimensions: 10 m guard + 30 m x 250 µm x 0.25 µm) was used. The injection volume was 2 µL into a thermal desorption unit (TDU 2, Gerstel, Germany) (initial temperature 30 °C, after 1 min heated to 300 °C at a 720 °C/min rate; cooled injection system (CIS, Gerstel, Germany) initial temperature -20 °C after 1 min heated to 300 °C at a rate of 12 °C/s in splitless mode.

The temperature program of the GC oven started at 60 °C, was held for 1.5 min, increased by 30 °C/min to 159 °C and held for 4 min, then increased by 4 °C/min to 240 °C and held for 5 min, then increased by 5 °C/min to 259 °C and held for 4 min, then increased by 5 °C/min to 300 °C and held for 15.45 min.

# Quality Assurance and Quality Control

Method Detection Limits (MDL) and Method Quantification Limits (MQL) for the total extraction of biota tissue (JenII), extracts from sampling of SPM with coated jars (CJ) and extracts of PSDs in water (PSD) were calculated as follows:

| $MQL\text{ }\text{ =mean(}C_{Blank})\times10\times standard deviation(C_{Blank})$ | (1) |
| --- | --- |
| $MDL\text{ }\text{ =mean(}C_{Blank})\times3\times standard deviation(C_{Blank})$ | (2) |

Table S2: Analyzed compounds with corresponding compound groups, log K_OW_, K_Lip:DC_ (for the polymer coated on jars), K_Lip:SSP_ (for the polymer used in water sampling), log K_DC:water_ and the MQL values of the extraction method of biota tissue (JenII), coated jars (CJ) and PSDs in water (PSD).

| **Compound** | **Compound Group** | **log *K*****_OW_^[[1]](#footnote-1)^** | ***K_L_*_ip:DC_ (kg/kg)****^[[2]](#footnote-2)^** | ***K_L_*_ip:SSP_ (kg/kg)**1 | **log *K*_SSP:water_****^[[3]](#footnote-3)^** | **log *K*_SSP:water_ SE**3 | **Molar Mass** | **MQL Jen II (pg/µL)** | **MQL CJ (pg/µL)** | **MQL PSD (pg/µL)** |
| --- | --- | --- | --- | --- | --- | --- | --- | --- | --- | --- |
| Acenaphthylene | PAH | 3.80 | 2.98* | 7.4 | 3.04 |  | 290.80 | <0.1 | 1.78 | <0.1 |
| Acenaphthene | PAH | 3.97 | 4.10* | 10.2 | 3.43 | 0.05 | 152.20 | 75.69 | 5.45 | 48.4 |
| Anthracene | PAH | 4.63 | 6.89* | 17.1 | 3.91 | 0.05 | 154.21 | <0.1 | 7.44 | 0.1 |
| Benzo(a)anthracene | PAH | 5.83 | 22.40* | 55.6 | 4.86 | 0.05 | 166.22 | <0.1 | 3.45 | 6.11 |
| Benzo(a)pyrene | PAH | 5.99 | 35.05* | 87 | 5.26 | 0.07 | 228.29 | 22.93 | 5.17 | <0.1 |
| Benzo(b)fluoranthene | PAH | 5.83 | 37.07* | 92 | 5.33 | 0.04 | 252.32 | 22.8 | 13.1 | 14.9 |
| Benzo(ghi)perylene | PAH | 6.60 | 51.57* | 128 | 5.61 | 0.05 | 252.32 | <0.1 | 1.15 | <0.1 |
| Benzo(k)fluoranthene | PAH | 5.85 | 30.58* | 75.9 | 5.29 | 0.06 | 252.32 | <0.1 | 7.1 | 7.2 |
| Chrysene | PAH | 5.67 | 19.74* | 49 | 4.84 | 0.07 | 278.35 | <0.1 | 2.92 | 11.53 |
| Dibenzo(a,h)anthracene | PAH | 6.51 | 59.23* | 147 | 5.70 | 0.08 | 276.34 | <0.1 | 1.9 | 10.45 |
| Fluoranthene | PAH | 4.98 | 12.21* | 30.3 | 4.28 | 0.07 | 276.34 | 31.86 | 4.12 | 25.92 |
| Fluorene | PAH | 4.14 | 5.16* | 12.8 | 3.56 | 0.05 | 257.54 | 10.53 | 1.1 | <0.1 |
| Indeno(1,2,3-cd)pyrene | PAH | 6.53 | 50.36* | 125 | 5.60 | 0.05 | 291.99 | <0.1 | 4.6 | <0.1 |
| Naphthalene | PAH | 3.40 | 2.98* | 7.4 | 2.8 | 0.04 | 326.43 | 855.48 | 49.65 | >1000 |
| Phenanthrene | PAH | 4.49 | 8.38* | 20.8 | 3.82 | 0.07 | 395.32 | <0.1 | 4.14 | <0.1 |
| Pyrene | PAH | 5.06 | 13.17* | 32.7 | 4.39 |  | 498.66 | 32.09 | 4.51 | 14.36 |
| PCB 101 | PCB | 6.38 | 11.93 | 22.1 | 5.99 | 0.06 | 290.83 | <0.1 | 2.27 | <0.1 |
| PCB 118 | PCB | 6.74 | 14.65 | 30.4 | 6.11 |  | 380.91 | <0.1 | 1.23 | 0.41 |
| PCB 138 | PCB | 6.83 | 12.36 | 26.6 | 6.49 | 0.03 | 284.78 | <0.1 | 1.27 | 2.03 |
| PCB 153 | PCB | 6.92 | 17.08 | 27.9 | 6.45 | 0.09 | 320.05 | <0.1 | 1.12 | 0.82 |
| PCB 180 | PCB | 7.36 | 19.66 | 31 | 6.76 | 0.09 | 354.49 | <0.1 | 1.95 | 9.04 |
| PCB 209 | PCB | 7.66 | 19.95 |  |  |  | 364.91 | 30.52 | 3.48 | 22.78 |
| PCB 28 | PCB | 5.67 | 8.64 | 12.2 | 5.25 | 0.06 | 318.03 | <0.1 | 1.78 | <0.1 |
| PCB 52 | PCB | 5.84 | 7.92 | 17.1 | 5.52 |  |  | <0.1 | 1.66 | <0.1 |
| alpha HCH | Pesticide | 3.78 | 11.72 | 29.1 | 3.04 |  |  | <0.1 | <0.1 | <0.1 |
| beta HCH | Pesticide | 3.84 | 86.22* | 214 | 1.94 |  |  | <0.1 | 3.52 | 1.2 |
| DDD | Pesticide | 6.22 | 22.00* | 54.6 | 4.98 |  |  | <0.1 | <0.1 | 3.22 |
| DDE | Pesticide | 6.95 | 7.17* | 17.8 | 6.04 |  |  | <0.1 | <0.1 | 1.47 |
| DDT | Pesticide | 6.22 | 14.71* | 36.5 | 5.79 |  |  | 3.45 | 0.59 | <0.1 |
| delta HCH | Pesticide | 4.14 | 53.59* | 133 | 2.37 |  |  | 1.3 | 3.35 | <0.1 |
| Dicofol | Pesticide |  |  |  |  |  |  | <0.1 | 2.22 | <0.1 |
| gamma HCH | Pesticide | 3.69 | 8.28 | 33.5 | 2.99 |  |  | <0.1 | 4.19 | <0.1 |
| HCB | Pesticide | 5.73 | 8.98 | 11.8 | 4.92 |  |  | <0.1 | 2.09 | <0.1 |
| Acenaphthylene d8 | PRC | 3.80 | 4.35* | 10.8 | 3.04 |  |  |  |  | <0.1 |
| Anthracene d10 | PRC | 4.63 | 7.13* | 17.7 | 3.91 |  |  |  |  | <0.1 |
| Fluoranthene d10 | PRC | 4.98 | 12.21* | 30.3 | 4.28 |  |  |  |  | <0.1 |
| PCB 10 | PRC | 4.84 | 3.72* | 9.24 | 4.35 |  | 188.29 |  |  | <0.1 |
| PCB 104 | PRC | 5.81 | 5.24* | 13 | 5.88 | 0.08 | 223.09 |  |  | <0.1 |
| PCB 30 | PRC | 5.44 | 4.97* | 12.3 | 5.01 |  | 212.31 |  |  | <0.1 |

# Passive Sampling of Water

The PRCs used were the deuterated PAHs Acenaphthylene d8, Anthracene d10, Fluoranthene d10 and the three PCB congeners 10, 30 and 104 that were not contained in commercial mixtures. Spiking was conducted as described by Smedes and Booji [2], by forcing the PRCs from a pure methanol solution into the silicone sheets by gradually diluting the solution with water over a time range of 7 days, until the fraction of methanol was 50% v/v.


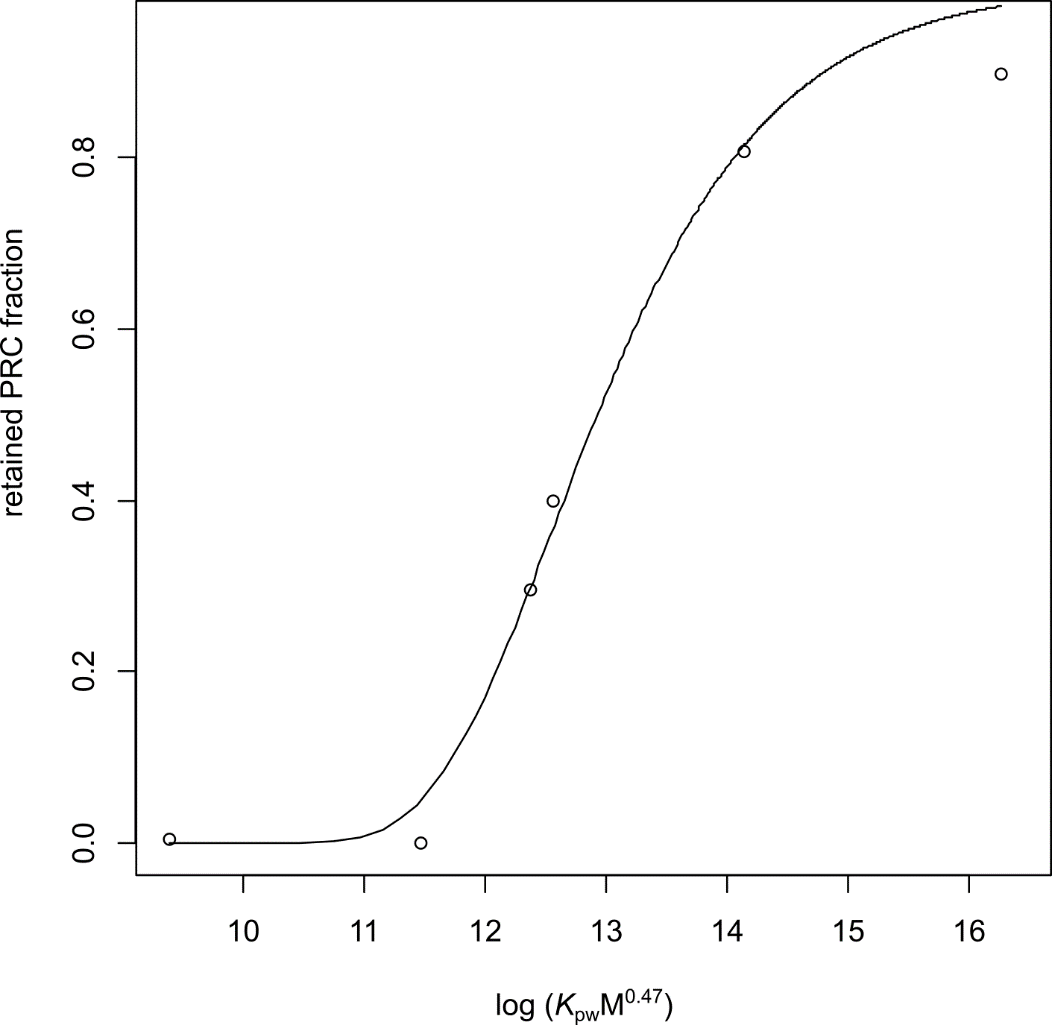


Figure S2: Example of a nls-model for the fraction of retained PRCs as a function of log (K_pw_M^0.47^) fitted to data from SSP sampler Nr. 2.

Table S3: Modelling results for the four SSP samplers deployed in water, with the estimates for B, the corresponding standard error and the estimates for R_S_ for DDE as an example compound.

| **Sampler Nr.** | **Estimate for B** | **Std. Error for B** | **R_S_ for DDE (molar mass 318.03)** |
| --- | --- | --- | --- |
| 1 | 52.393 | 5.138 | 3.49 |
| 2 | 58.253 | 4.566 | 3.88 |
| 3 | 54.518 | 2.957 | 3.63 |
| 4 | 50.62 | 3.75 | 3.74 |

# Passive Sampling of SPM

Partition coefficients between lipid and the silicone used for coating the jars (Dowsil DC 1-2577) *K*_Lip:DC_ provided by Gilbert, Witt [3] were not available for all compounds used in this study. A mean conversion factor *K*_DC:SSP_= 2.482±0.77 was calculated [3]. Missing *K*_Lip:DC_=*K*_Lip:SSP_/2.482 were calculated, *K*_Lip:SSP_ were taken from Smedes et al. [1].

In order to prevent using data that might be impacted by depletion of the SPM phase by the silicone of the coated jars, the following exclusion criteria were applied: assuming the mass of the organic carbon (m_OC_) is the main sorption phase of HOCs in the SPM, the following criteria were adapted from [4]:

| $0.05\text{ }\text{ <}\frac{m_{Sil}\times K_{DC/OC}}{m_{OC}}$ | (3) |
| --- | --- |

Where m_Sil_ is the silicone mass in a coated jar (DC 1-2577) and *K*_DC/OC_ is a silicone/organic carbon partition coefficient taken from Niu et al. [5]. The depletion criterion was calculated for each compound in each coated jar, and samples exceeding 0.05 were excluded from further analysis. In addition, some outliers were removed, since some samples showed very high specific concentrations, probably because they originated from two different manufacturing batches. See table S9 for which thicknesses were used with sample and compound.

# Stable Isotope Analysis

To prepare samples for stable isotope analysis, homogenized muscle tissue samples were freeze-dried, ground by hand using a ceramic mortar and pestle and weighed into tin capsules (3.5 mm × 5 mm, HEKAtech, Germany) (~ 1 mg). Samples were analyzed in triplicates. A EuroEA3000 elemental analyzer (HEKAtech, Germany) was used for the combustion of the samples to N_2_ and CO_2_ gas. Flash combustion was performed with a 10 mL O_2_ pulse in a commercially available combustion reactor filled with wolfram oxide, and silver cobalt oxide (HE46820995, HEKAtech, Germany) kept at 1050°C. The formed combustion product gases were swept with the helium carrier gas (gas flow = 80 mL/min) through a reduction furnace filled with copper kept at 650 °C. The remaining water was trapped with phosphorus pentoxide. Afterward, the gases were separated isothermally at 70 °C on a GC column (HE26070500, HEKAtech, Germany) and transferred into the IRMS via a ConFloIV open split system. Isotope ratios of an element (E) were expressed as delta notation (δ^13^C or δ^15^N) in parts per thousand (‰) relative to the international standards (Eq. 4). The international standards were VPDB (Vienna Pee Dee Belemnite) for carbon and air for nitrogen, respectively.

| $\delta E_{sample} = \left( \frac{R_{sample}}{R_{standard}}-1 \right)\times1000$ | (4) |
| --- | --- |

R_sample_ and R_standard_ are the isotope ratio (^13^C/^12^C or ^15^N/^14^N) of the sample or the corresponding ratio of the standard, respectively. Normalization of measured isotope compositions to international isotope-delta scales was done by analyzing reference materials in the same way as the samples and then applying a two-point calibration approach. The international reference materials used for normalization were: IAEA-CH6 (-10.45‰), IAEA-CH7 (-32.15‰) and IAEA-CH3 (-24.72‰) for carbon and AS-3 (-0.35‰) and AS-11 (+11.0‰) for nitrogen. The analytical precision (SD of multiple runs) was below ±0.2‰ for both δ^13^C and δ^15^N.

The invertebrate samples used as primary consumer references in this study were taken at the upstream sampling point near Priorau (see Figure S1) by kick sampling with a hand net (500 µm mesh width) from six subsampling sites at both shorelines. Individuals were sorted, identified and frozen on ice in the field.

The common implementation strategy for the water framework directive [6] suggests using mussels as the reference for TL=2, i.e., primary consumers. In the current study, we have used several primary consumers species as the reference (δ^15^N (mean±SD)) in 2017: 10.4±1.4‰ and 2020: 15.6±1.2‰, to consider benthic and pelagic nutrient pathways (Post 2007).

Table S4: Means and standard deviations of stable isotope data for carbon and nitrogen for all invertebrate species investigated in 2017.

| **Species** | **n** | **δ^13^C** | **d^15^N** |
| --- | --- | --- | --- |
| *Heptagenia* sp. | 6 | -28.33 ± 1.32 | 11.25 ± 0.56 |
| Chironomidae | 3 | -25.95 ± 1.02 | 12.75 ± 0.25 |
| *Sphaerium* ***sp.*** | 1 | -32.78 | 8.61 |
| *Radix balthica* | 10 | -25.26 ± 1.72 | 11.24 ± 1.23 |
| *Heptagenia sulphurea* | 1 | -30.75 | 10.41 |
| *Dreissena polymorpha* | 3 | -30.88 ± 2.55 | 11.34 ± 1.99 |
| Tipuliidae | 5 | -25.35 ± 1.09 | 9.13 ± 1.94 |

Table S5: Means and standard deviations of stable isotope data for carbon and nitrogen for all invertebrate species investigated in 2020.

| **Species** | **n** | **d^13^C** | **d^15^N** |
| --- | --- | --- | --- |
| Chironomidae | 1 | -26.59 | 15.37 |
| *Heptagenia flava* | 2 | -26.23 ± 0.05 | 17.12 ± 0.04 |
| *Corbicula fluminea* | 1 | -28.91 | 14.91 |
| *Dreissena polymorpha* | 2 | -28.75 ± 0.04 | 14.57 ± 0.15 |


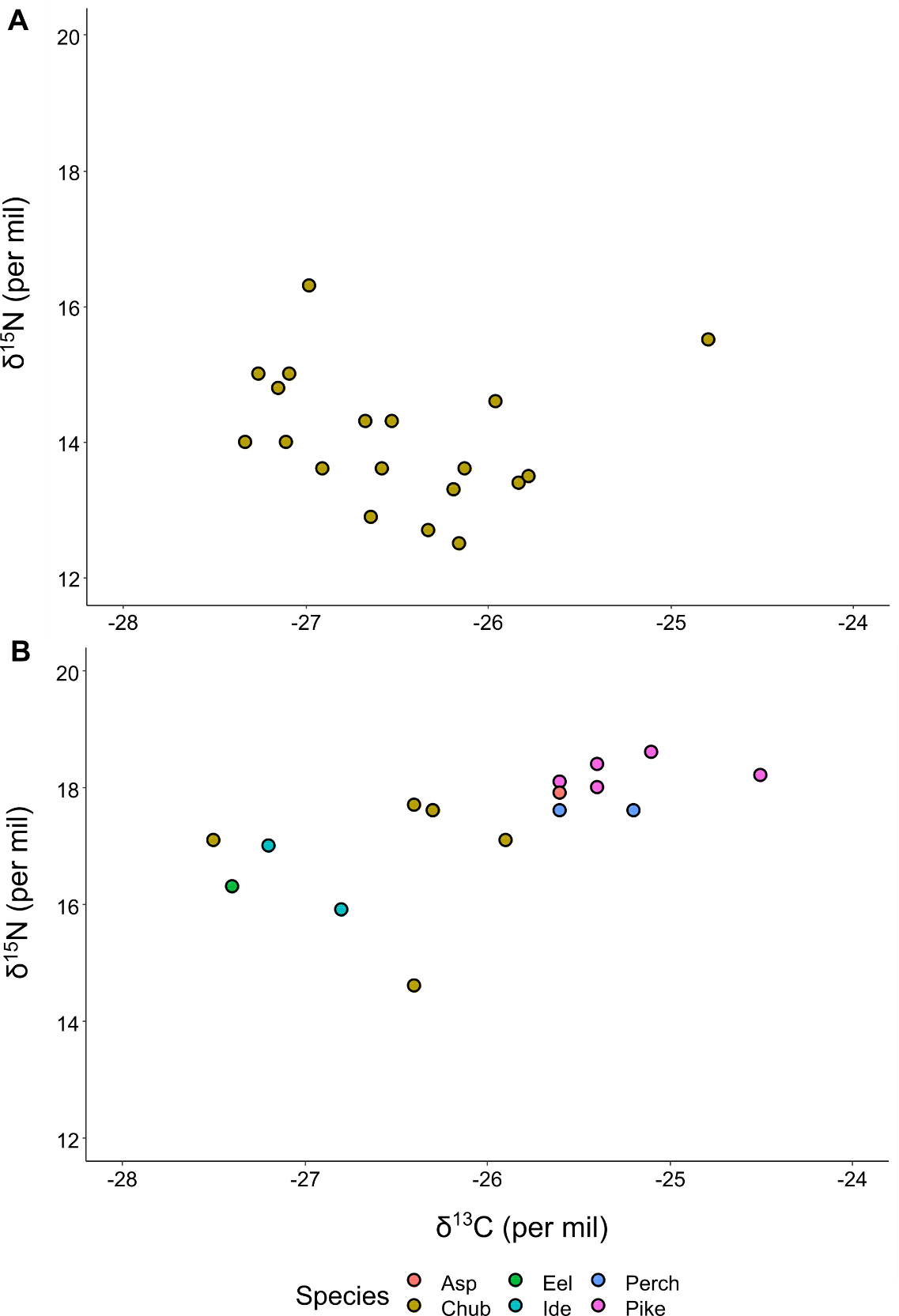


Figure S3: Plot of δ^15^N against δ^13^C for chub (2017) (A) and fish species from 2020 (B).

# Partitioning between Water and SPM

Table S6: List with mean and standard deviations (sd) equilibrium model lipid concentrations derived from PSD sampling of water and SPM.

| **Compound** | **log *K*_OW_** | **mean *C*_Lip⇆SPM_ (pg/mg)** | **sd *C*_Lip⇆SPM_ (pg/mg)** | **mean *C*_Lip⇆Water_ (pg/mg)** | **sd *C*_Lip⇆Water_ (pg/mg)** |
| --- | --- | --- | --- | --- | --- |
| beta HCH | 3.84 | 5660.36 | 2190.95 | 67.48 | 6.08 |
| Acenapthene | 3.97 | 227.19 | 95.03 | 20.07 | 0.66 |
| Fluorene | 4.14 | 425.31 | 78.02 | 60.20 | 0.94 |
| delta HCH | 4.14 | 1023.63 | 584.16 | 34.89 | 4.13 |
| Phenanthrene | 4.49 | 3272.79 | 635.90 | 454.28 | 5.68 |
| Anthracene | 4.63 | 1141.15 | 336.29 | 31.50 | 16.91 |
| Fluoranthene | 4.98 | 3308.90 | 1104.70 | 276.62 | 8.47 |
| Pyrene | 5.06 | 4102.87 | 1893.32 | 532.72 | 9.73 |
| PCB 28 | 5.67 | 175.46 | 60.94 | 6.12 | 0.17 |
| Chrysene | 5.67 | 3725.99 | 1315.86 | 406.07 | 46.17 |
| HCB | 5.73 | 1768.14 | 57.96 | 243.56 | 4.40 |
| Benzo(a)anthracene | 5.83 | 3250.81 | 1170.87 | 158.05 | 40.31 |
| Benzo(b)fluoranthene | 5.83 | 5440.90 | 1228.29 | 1354.95 | 137.78 |
| PCB 52 | 5.84 | 177.33 | 68.77 | 28.05 | 1.72 |
| Benzo(k)fluoranthene | 5.85 | 7609.78 | 3420.57 | 260.67 | 126.46 |
| Benzo(a)pyrene | 5.99 | 2916.38 | 750.30 | 234.67 | 16.35 |
| DDD | 6.22 | 23860.48 | 9033.42 | 2133.28 | 66.91 |
| DDT | 6.22 | 4889.83 | 0.00 | 2531.00 | 932.59 |
| PCB 101 | 6.38 | 606.34 | 0.00 | 110.20 | 7.50 |
| Benzo(ghi)perylene | 6.60 | 5855.20 | 2774.73 | 927.42 | 62.56 |
| PCB 118 | 6.74 | 2777.97 | 0.00 | 115.26 | 4.56 |
| PCB 138 | 6.83 | 2922.34 | 0.00 | 729.85 | 44.23 |
| PCB 153 | 6.92 | 1565.29 | 793.08 | 680.41 | 26.21 |
| DDE | 6.95 | 8392.30 | 3107.83 | 2451.72 | 26.31 |


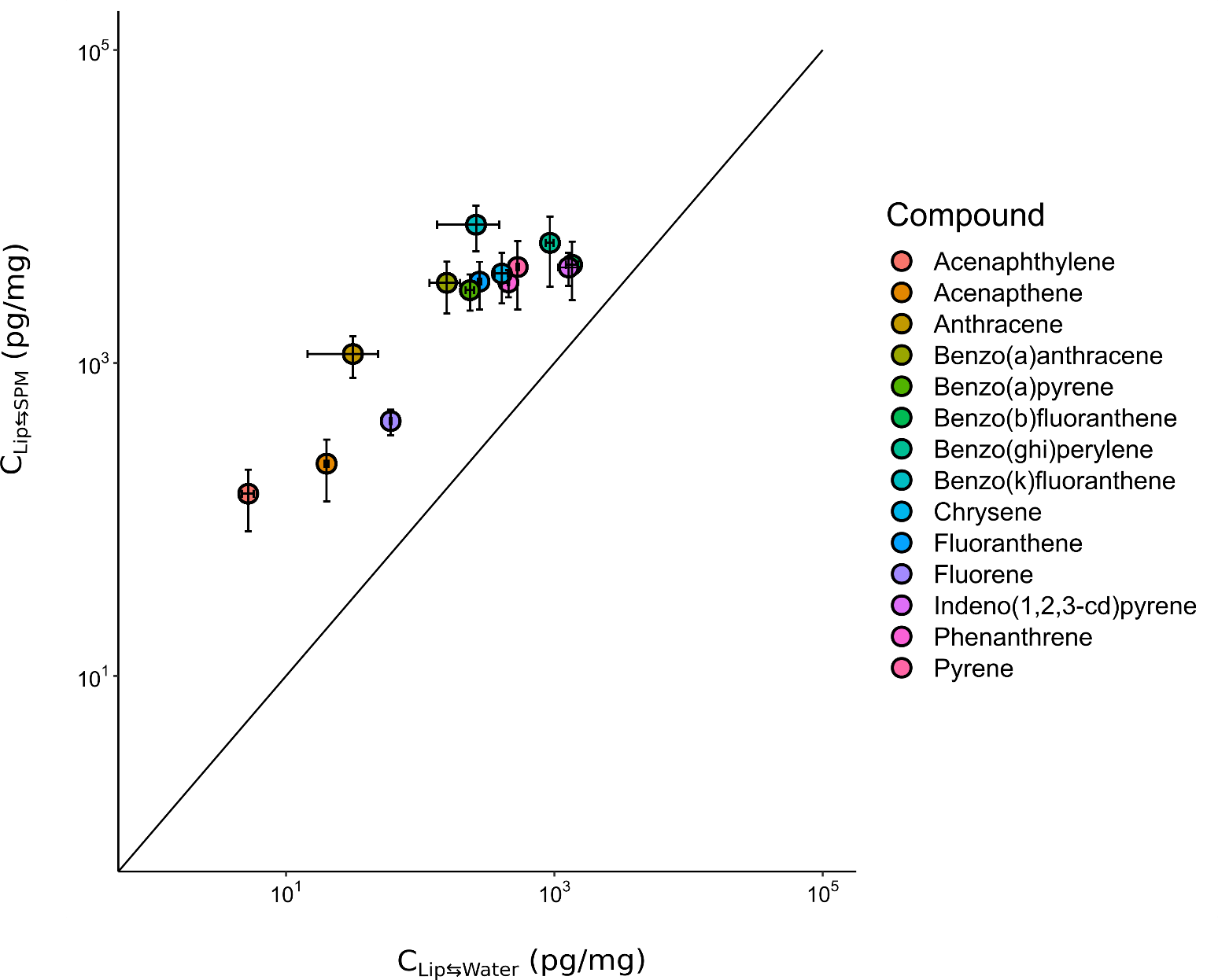


Figure S4 Mean C_Lip⇆SPM_ against mean C_Lip⇆Water_ for PAHs. The diagonal line symbolizes the 1:1 ratio. Error bars represent the standard deviation of the mean.


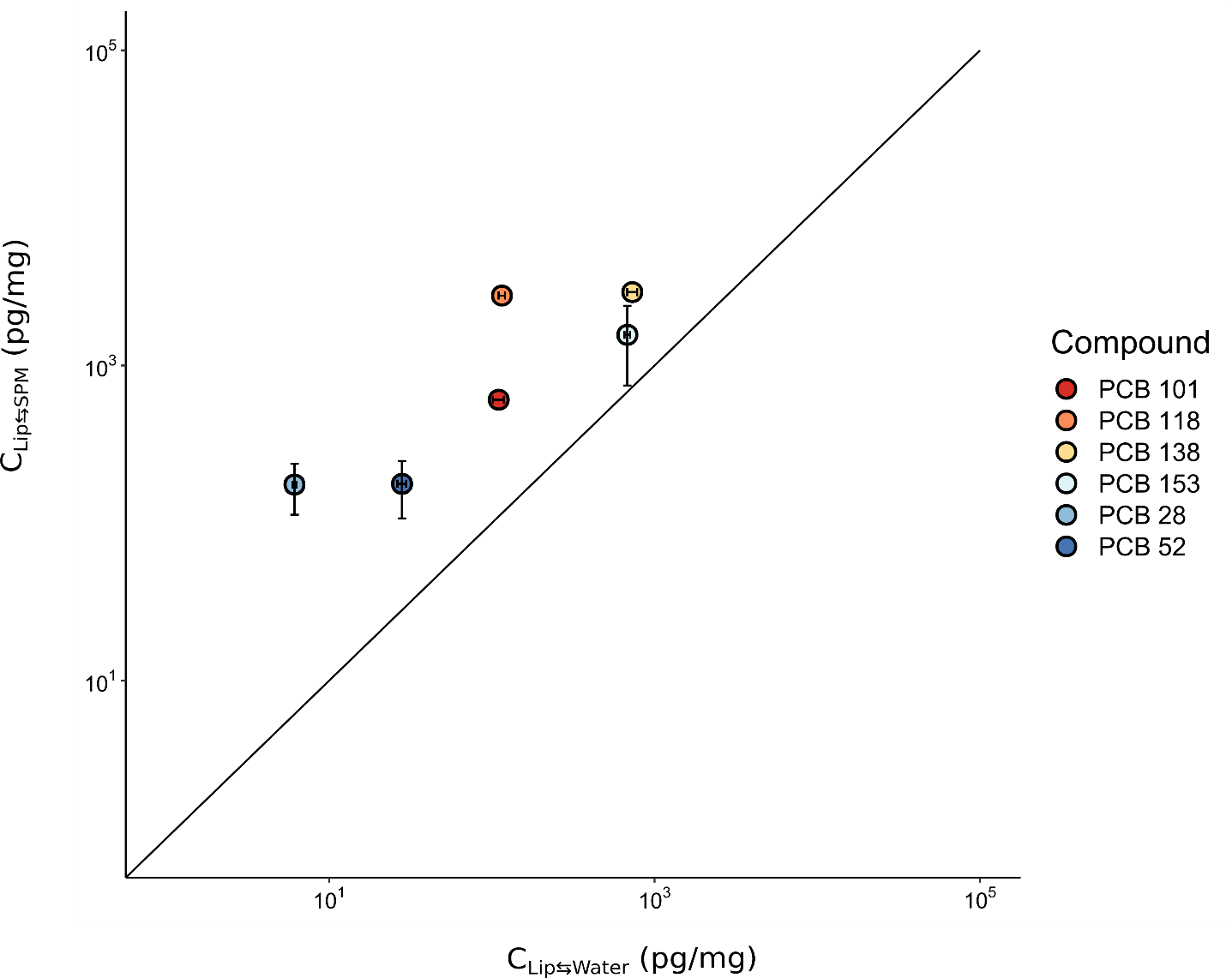


Figure S5: Mean C_Lip⇆SPM_ against mean C_Lip⇆Water_ for PCBs. The diagonal line symbolizes the 1:1 ratio. Error bars represent the standard deviation of the mean.


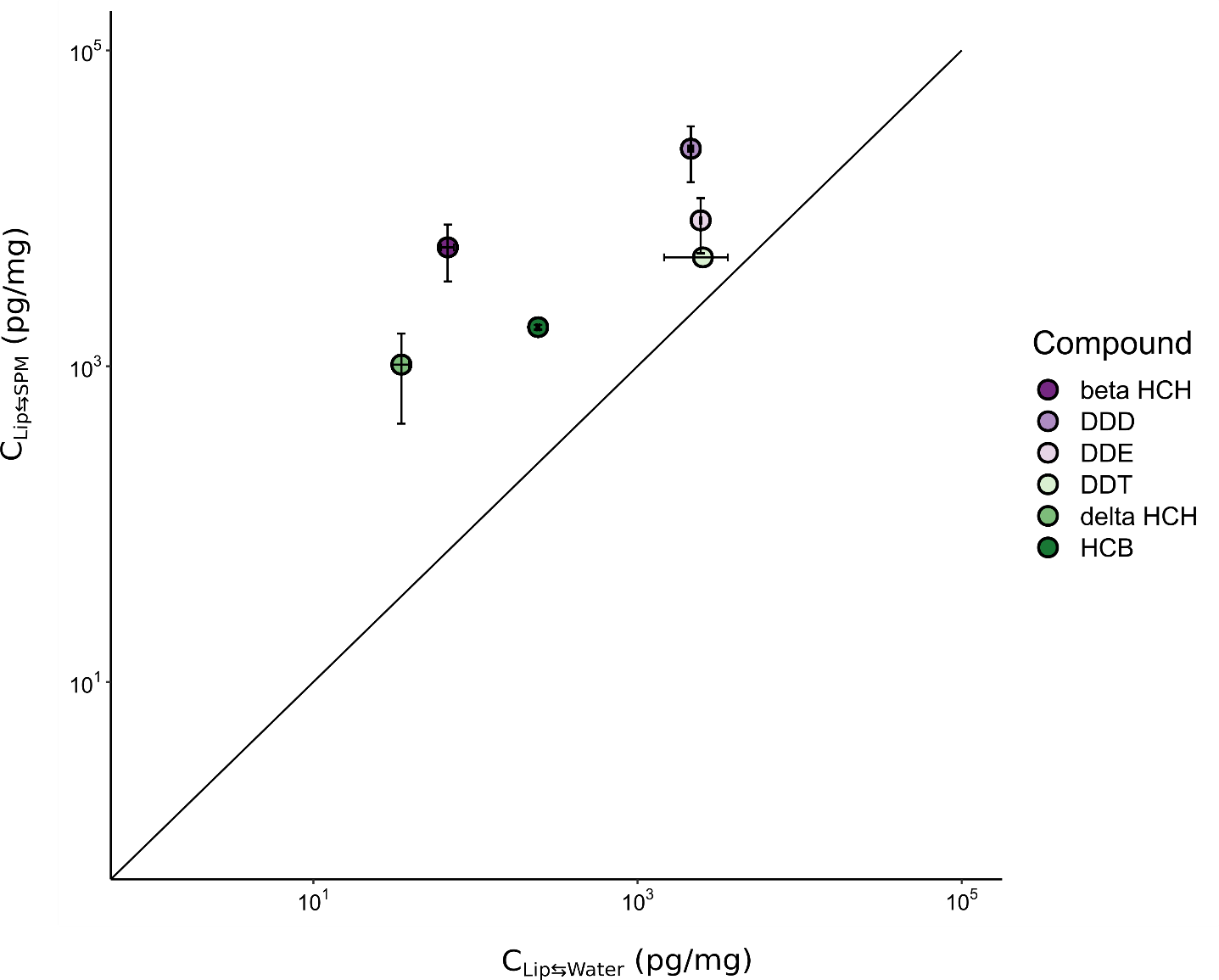


Figure S6: Mean C_Lip⇆SPM_ against mean C_Lip⇆Water_ for OCPs. The diagonal line symbolizes the 1:1 ratio. Error bars represent the standard deviation of the mean.

**
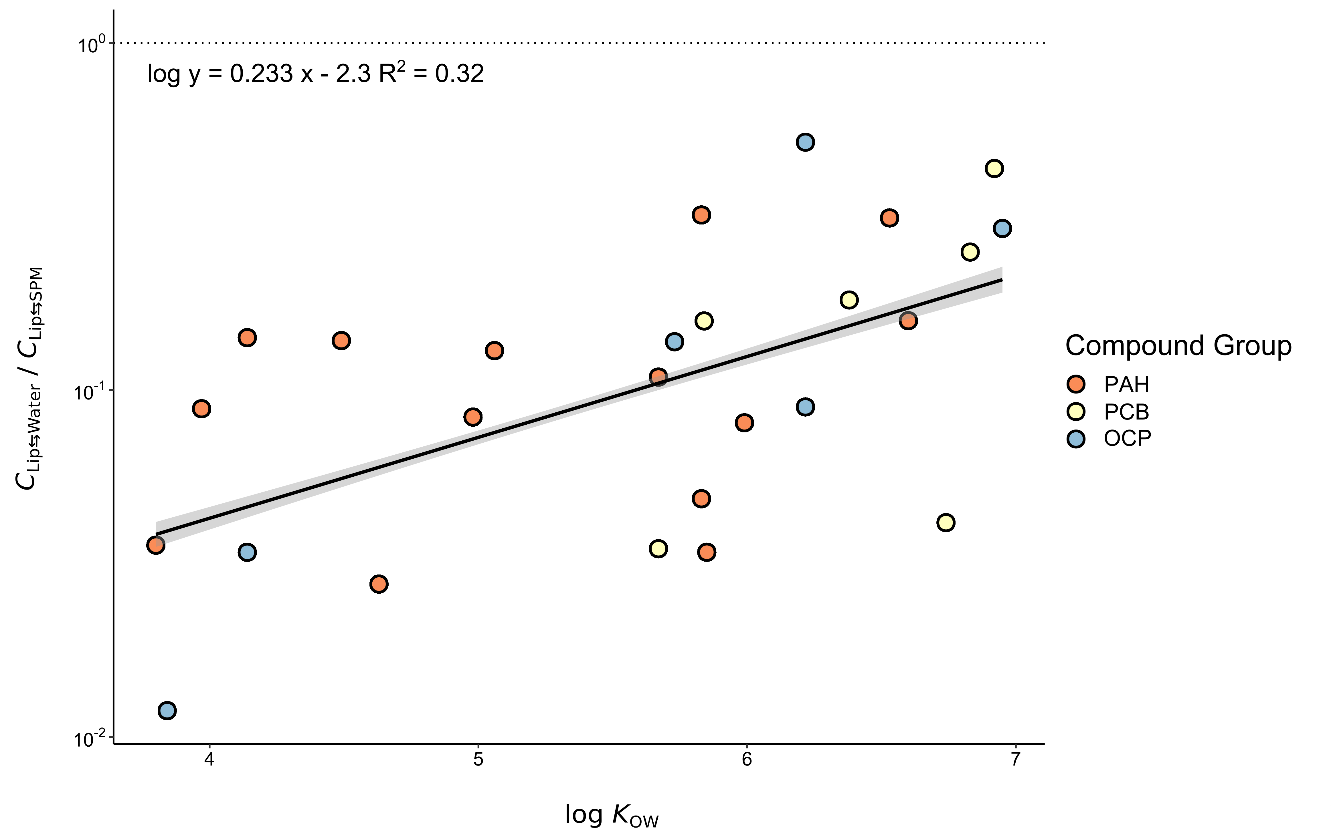
**

Figure S7: Ratio between C_Lip⇆Water_ and C_Lip⇆SPM_ plotted against the log K_OW_ of each individual chemical. The dotted line marks the ratio = 1. A linear model is given as an equation and as a black line, along with the grey band illustrating the 95% confidence interval.

# Additional Results for Fish


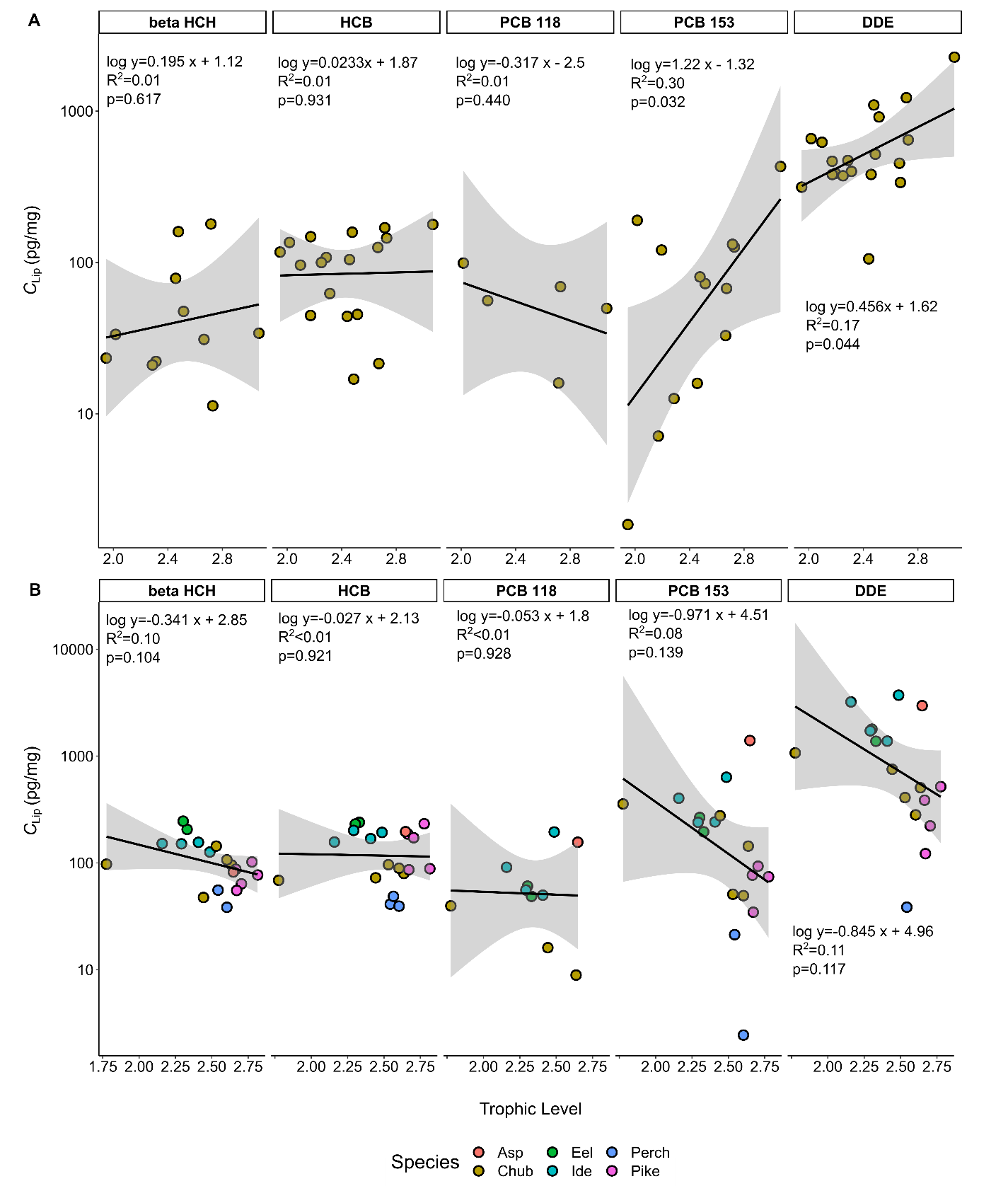


Figure S8: Lipid-normalized concentrations in the investigated fish samples plotted against trophic level of (A) individuals of European chub sampled in 2017 and (B) all fish species (catfish was excluded) sampled in 2020 for the model compounds beta HCH, HCB, PCB 118, PCB 153 and DDE spanning a wide range of hydrophobicities. A log-linear model is plotted as an equation in each panel and as a black line, along with the grey band illustrating the 95% confidence intervals.


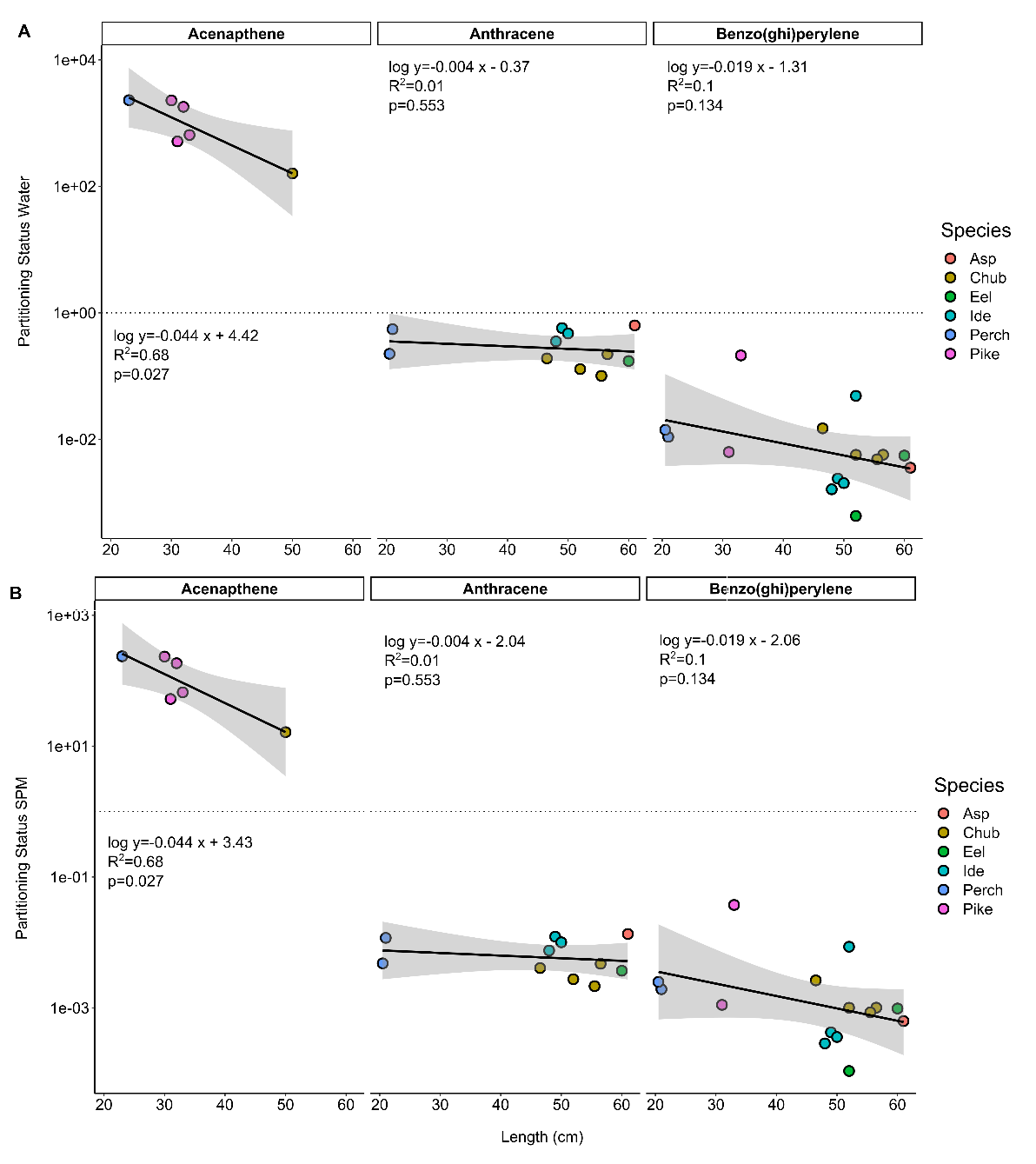


Figure S9: Plot of the mean partitioning status of Acenaphthylene, Anthracene and Benzo(ghi)perylene for water (A) and SPM (B) against the body length of eel, ide, perch, chub, pike and asp sampled in 2020. A log-linear model is plotted as an equation in each panel and as a black line, along with the grey band illustrating the 95% confidence interval.

The relative weight W_rm_ with parameters specific for each fish species was calculated according to Froese [8]:

| $Wrm\text{ }\text{ =100×}\frac{W}{a_{m}L^{b_{m}}}$ | (5) |
| --- | --- |

Where W is the weight in grams, L is the length in cm and a_m_ and b_m_ species-specific parameters taken from Froese and Pauly [9] and are listed in Table S7.

Table S7: Species specific model parameters taken from FishBase.

| **Species** | **a_m_** | **b_m_** |
| --- | --- | --- |
| Eel | 0.00095 | 3.17 |
| Ide | 0.00759 | 3.13 |
| Perch | 0.01 | 3.08 |
| Chub | 0.00776 | 3.12 |
| Pike | 0.00437 | 3.09 |
| Catfish | 0.00813 | 2.98 |
| Asp | 0.00759 | 3.12 |


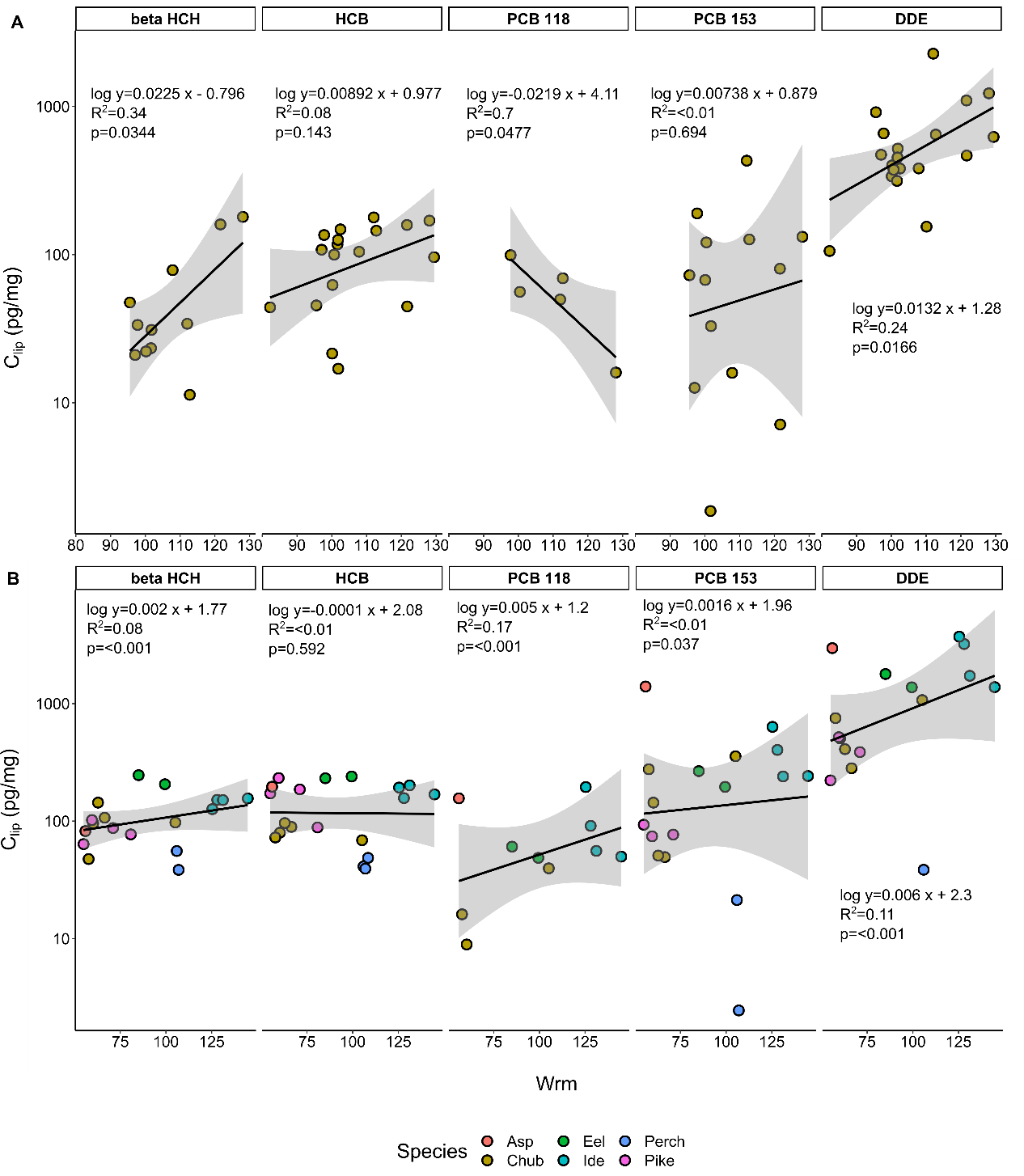


Figure S10: Lipid-normalized concentrations plotted against relative weight W_rm_ according to Froese [8] of (A) individuals of European chub sampled in 2017 and (B) all fish species (catfish was exclude) sampled in 2020 for the compounds beta HCH, HCB, PCB 118, PCB 153 and DDE. A log-linear model is plotted as an equation in each panel and as a black line, along with the grey band illustrating the 95% confidence interval.


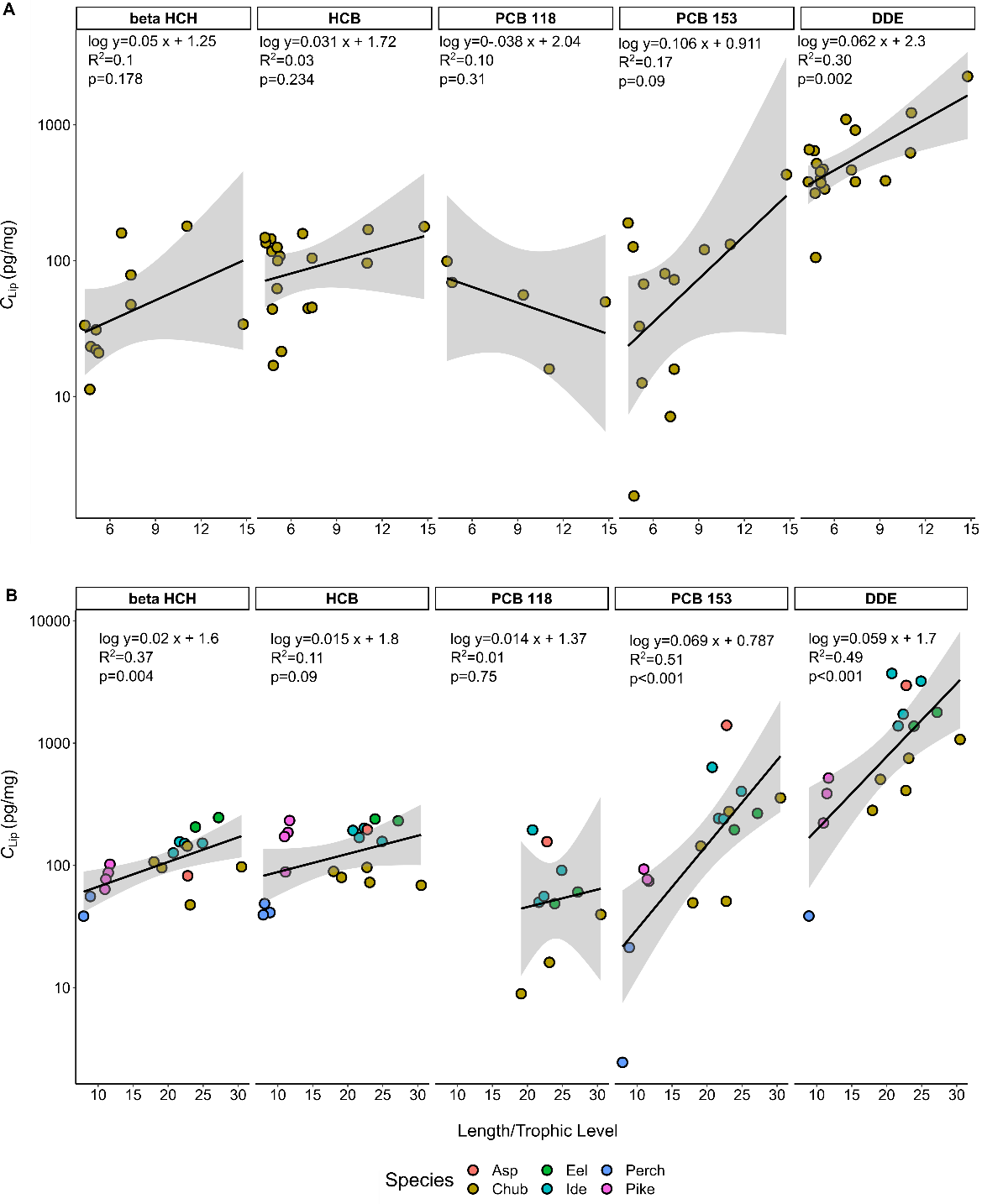


Figure S11: Lipid-normalized concentrations plotted against TL-normalized body length of (A) individuals of European chub sampled in 2017 and (B) all fish species (catfish was excluded from the analysis) sampled in 2020 for the compounds beta HCH, HCB, PCB 118, PCB 153 and DDE. A log-linear model is plotted as an equation at the top of each plot and as a black line, along with the grey band illustrating the 95% confidence interval.

# References

1. Smedes, F., et al., *Partitioning of hydrophobic organic contaminants between polymer and lipids for two silicones and low density polyethylene.* Chemosphere, 2017. **186**: p. 948-957.

2. Smedes, F. and K. Booji, *Guidelines for passive sampling of hydrophobic contaminants in water using silicone rubber samplers.* ICES Techniques in Marine Environmental Sciences, 2012. **52**: p. 20.

3. Gilbert, D., et al., *Polymers as Reference Partitioning Phase: Polymer Calibration for an Analytically Operational Approach To Quantify Multimedia Phase Partitioning.* Analytical Chemistry, 2016. **88**(11): p. 5818-5826.

4. Mayer, P., et al., *Equilibrium sampling devices.* Environmental Science & Technology, 2003. **37**(9): p. 184A-191A.

5. Niu, L., et al., *Mixture Risk Drivers in Freshwater Sediments and Their Bioavailability Determined Using Passive Equilibrium Sampling.* Environmental Science & Technology, 2020. **54**(20): p. 13197-13206.

6. Deutsch, K.A., et al., *COMMON IMPLEMENTATION STRATEGY FOR THE WATER FRAMEWORK DIRECTIVE (2000/60/EC). Guidance Document No. 32 ON BIOTA MONITORING (THE IMPLEMENTATION OF EQSBIOTA) UNDER THE WATER FRAMEWORK DIRECTIVE.* CTIT technical reports series, 2014.

7. Post, D.M., *USING STABLE ISOTOPES TO ESTIMATE TROPHIC POSITION: MODELS, METHODS, AND ASSUMPTIONS.* Ecology, 2002. **83**(3): p. 703-718.

8. Froese, R., *Cube law, condition factor and weight–length relationships: history, meta-analysis and recommendations.* Journal of Applied Ichthyology, 2006. **22**(4): p. 241-253.

9. Froese, R. and D. Pauly. *FishBase*. 2019 [cited 2021 03.02.2021]; Available from: [www.fishbase.org](file:///C:\Users\brauns\Nextcloud\Publikationen\Muldeprojekt\Theo%20Wernicke\02%20revision\www.fishbase.org).

1. Adapted from Smedes et al. 2017 [↑](#footnote-ref-1)
2. Adapted from Gilbert et al. 2016 or *calculated as described in section 6. [↑](#footnote-ref-2)
3. Adapted from Smedes et al. 2019 [↑](#footnote-ref-3)
